# Ice Recrystallization Inhibition by Amino Acids: The Curious Case of Alpha- and Beta-Alanine

**DOI:** 10.1021/acs.jpclett.1c04080

**Published:** 2022-03-03

**Authors:** Matthew T. Warren, Iain Galpin, Fabienne Bachtiger, Matthew I. Gibson, Gabriele C. Sosso

**Affiliations:** † Department of Chemistry, University of Warwick, Gibbet Hill Road, Coventry CV4 7AL, United Kingdom; ‡ Warwick Medical School, University of Warwick, Gibbet Hill Road, Coventry CV4 7AL, United Kingdom

## Abstract

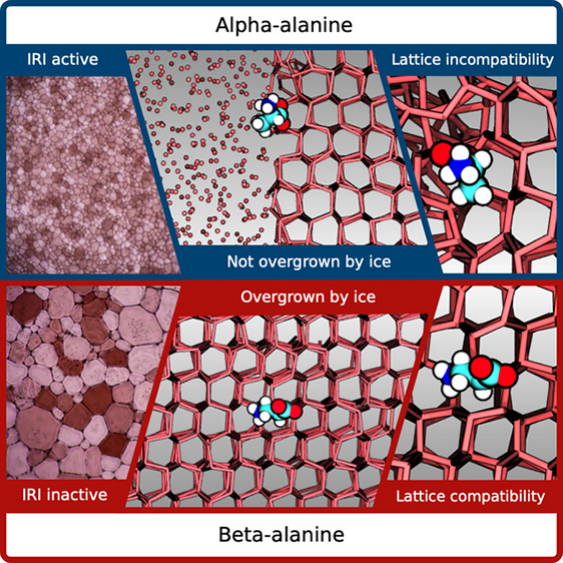

Extremophiles produce macromolecules which inhibit ice recrystallization, but there is increasing interest in discovering and developing small molecules that can modulate ice growth. Realizing their potential requires an understanding of how these molecules function at the atomistic level. Here, we report the discovery that the amino acid l-α-alanine demonstrates ice recrystallization inhibition (IRI) activity, functioning at 100 mM (∼10 mg/mL). We combined experimental assays with molecular simulations to investigate this IRI agent, drawing comparison to β-alanine, an isomer of l-α-alanine which displays no IRI activity. We found that the difference in the IRI activity of these molecules does not originate from their ice binding affinity, but from their capacity to (not) become overgrown, dictated by the degree of structural (in)compatibility within the growing ice lattice. These findings shed new light on the microscopic mechanisms of small molecule cryoprotectants, particularly in terms of their molecular structure and overgrowth by ice.

The cryopreservation of biological materials is a key factor in regenerative medicine and cell-based therapies,^1,2^ but strategies are required to limit the cellular damage incurred at subzero temperatures.^3^ Organic solvents such DMSO and glycerol are widely used as cryoprotectants,^4^ and while they are effective, they are not suitable for all cell types and not all cells are typically recovered post-thaw.^5,6^ The cryopreservation of large tissues also remains extremely challenging. A major contributor to post-thaw damage is ice recrystallization (IR), an Ostwald ripening process^7^ whereby large ice crystals grow in favor of smaller ones, exerting mechanical and osmotic stress on the sample which often leads to post-thaw cell death. Ice recrystallization inhibition (IRI) represents an appealing strategy to improve cryopreservation outcomes, and ice recrystallization inhibitors (IRIs) have consequently received significant interest in recent years. However, the mechanism(s) by which these molecules inhibit IR and the underlying molecular determinants are poorly understood. In turn, this hampers the discovery of new materials that possess a sought-after balance of potent inhibition (at low concentrations), biocompatibility, and amenability to low cost, large scale production.

The IRI-active materials discovered to date are diverse, ranging from polymers,^8^ to the well-known ice-binding proteins,^9^ to small molecules.^10^ These materials can all give rise to the same macroscopic effect defined as IRI, but multiple molecular-level mechanisms appear to underpin the observable phenomenon. For certain materials, including many of the antifreeze proteins (AFPs) and glycoproteins (AFGPs), IRI activity is linked to a molecule’s ability to bind to specific faces of an ice crystal, causing a local positive curvature of the ice front which inhibits further ice growth through the Gibbs–Thomson (Kelvin) effect.^11,12^ By arresting ice growth, surface adsorption also results in a non-colligative depression of the freezing point relative to the melting point, known as thermal hysteresis (TH). TH is often accompanied by dynamic ice shaping (DIS), whereby ice crystals display distinct morphologies reflecting the specific lattice face(s) onto which the AF(G)Ps adsorbed.^13^ The question remains: how do these materials first recognize and bind ice in a vast excess of liquid water? The answer is still unclear. A diversity of chemical motifs appear capable of binding to ice, but the relative contributions of hydrogen bonding, hydrophobic interactions,^14,15^ and ordered clathrate waters^16^ are still under debate.

Equally unresolved are the mechanistic details of IRI. Recent studies have confirmed that, although IRI and TH activities appear connected, the two properties are not directly correlated in AFPs.^17,18^ Meanwhile, for other IRI-active materials such as the small molecule carbohydrates reported by Ben and co-workers,^19,20^ there is no evidence of ice binding at all (i.e., no TH or DIS). These findings both point to an alternate mode of inhibition that is independent of ice binding and likely the primary mechanism of action for small molecules. One possible mechanism, proposed by Ben and colleagues,^19^ suggests that small molecules inhibit IR by disrupting the order of water in the interfacial region between bulk water and the preordered water layer surrounding an ice crystal. However, this hypothesis lacks extensive experimental support and is challenging to study computationally. Given that ice binding can often shape bound crystals into needlelike spicules that are injurious to biological materials, understanding this mode of inhibition represents a crucial step toward the identification of novel IR inhibitors which have clinical application. In this context, small molecules are also attractive targets because they can be efficiently manufactured, and obtaining structure–property relationships is simpler than for, e.g., polymers, which have intrinsic heterogeneity (e.g., dispersity).

Here, we report that the simple amino acid l-α-alanine (herein referred to as α-alanine) exhibits IRI activity at millimolar concentrations. We also show that its structural isomer, β-alanine, is IRI-inactive at equivalent concentrations, despite the structural similarity of these two forms. To unravel the origin of α-alanine’s IRI activity we used atomistic molecular dynamics (MD) simulations. These simulations allow for ice growth kinetics to be studied in the presence of (α/β-)alanine, shedding new light on the mechanisms of small molecule IRIs and the molecular determinants of IRI activity.

We began by assessing the ability of α- and β-alanine to inhibit IR using the “splat” cooling assay. This assay involves rapidly cooling a droplet of solution to form a polycrystalline ice monolayer (Figure 1c), which is then annealed at −8 °C. The growth of crystals within this layer is determined by comparing the mean grain size (MGS) after 30 min to a positive control for ice growth. A smaller relative (%) MGS value therefore indicates stronger inhibition. This assay requires salt (or other additives) to ensure a eutectic phase is formed, in order to avoid false positives that arise from using pure water alone.^21,22^ When α-alanine was tested in phosphate buffered saline (PBS), which is typically used for this assay, no IRI activity was observed (Figure S1). However, in a series of controls using α-alanine and betaine (another IRI-inactive small molecule of similar molecular weight to α-alanine), we found that including 10 mM NaCl led to significant IRI with α-alanine (and not betaine) and is a sufficient quantity of saline to avoid false positive hits (see the Supporting Information and Figure S1).^23^ We also found that α-alanine remains active in 10 mM phosphate buffer but not under higher concentrations of saline (100 mM NaCl) (Figure S1); hence, we suggest that the IRI activity of this system is sensitive to high concentrations of salt rather than specific components of the PBS solution. We also note that, while this assay is usually performed in PBS, the use of saline solution for IRI measurements is not uncommon in this field.^24,25^

**Figure 1:**
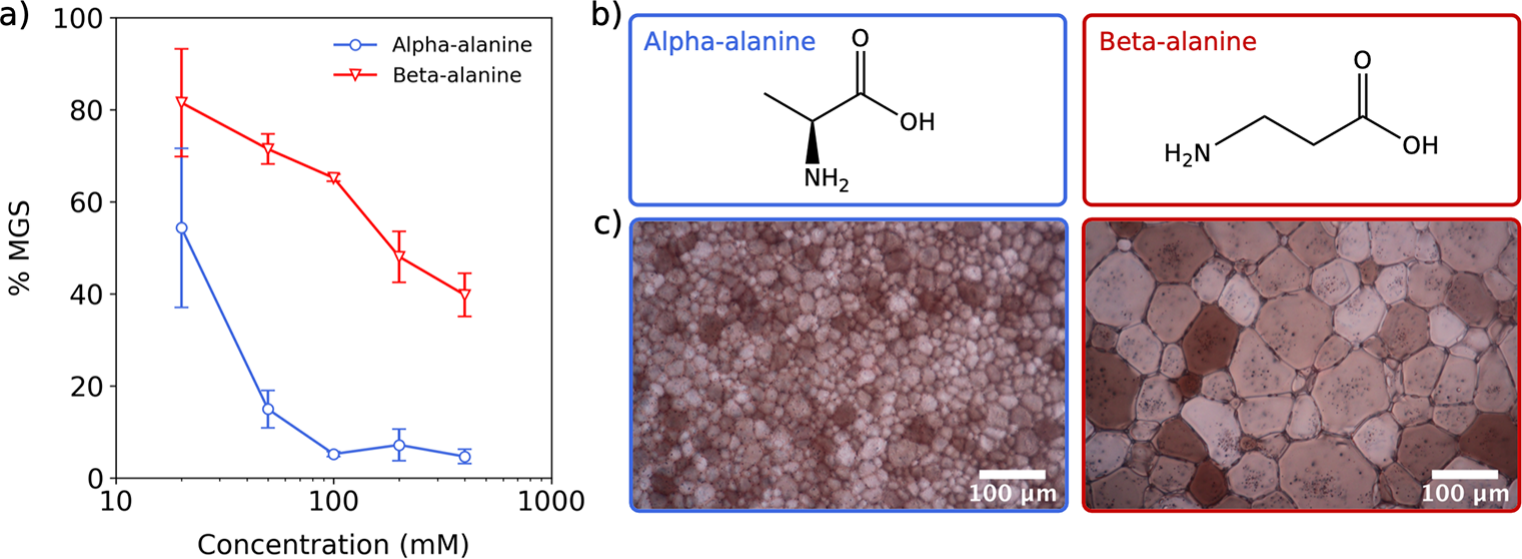
(a) Ice recrystallization inhibition activity of α-alanine and β-alanine. Error bars are ±1 SD from a minimum of three repeats. The percentage mean grain size (MGS) is reported relative to a saline control (10 mM NaCl). (b) Structures of α-alanine and β-alanine. (c) Example cryomicrographs of ice wafers from the “splat” cooling assay, grown in the presence of (50 mM) α-alanine or β-alanine.

Using 10 mM NaCl, we found that α-alanine is able to suppress ice growth almost entirely at millimolar concentrations, producing crystals which are dramatically smaller than in the controls (Figure 1a). This level of activity can be considered moderate in comparison to the most potent IRI-active materials, such as poly vinyl alcohol (PVA),^26^ but on the same magnitude as other small molecules tested under similar conditions.^24^ For example, PVA20 (where 20 is the degree of polymerization) achieves similar levels of inhibition to 100 mM α-alanine (∼10 mg/mL) at ∼1 mg/mL.^27^ However, the IRI activity of α-alanine must be considered in the context of the molecule’s size. Given that its structure comprises merely 13 atoms (corresponding to a molecular mass below 90 atomic mass units), to our knowledge α-alanine represents the smallest ice recrystallization inhibitor discovered to date; it is significantly smaller than other materials (including small molecules) reported elsewhere.^10,20,28^

In the knowledge that α-alanine is inactive at high salt concentrations (100 mM and above), we note that the cryoprotective applications of α-alanine specifically may be limited to cases where biological materials are stored in low-salt buffers. However, we highlight that there are a number bacterial and plant growth media which satisfy these minimal salt requirements (e.g., 2xYT,^29^ M9,^30^ TB,^31^ and MK^32^ media), and such materials require effective cryopreservation for both industrial and research applications.

To confirm whether this property is unique to α-alanine, we also tested the IRI efficacy of its structural isomer, β-alanine, noting that the stereoisomers l- and d-α-alanine are equally IRI active. Strikingly, despite the structural similarity to the α-form, the β-isomer does not exhibit any IRI activity at equivalent concentrations (Figure 1a). This difference was observed across a range of concentrations, most notably at 100 mM where the % MGS of α-alanine and β-alanine were 5% and 65%, respectively. It is important to highlight that almost any material can inhibit ice growth at sufficiently high concentrations (e.g., as shown for β-alanine in Figure 1a). However, given that α-alanine demonstrates a clear inhibitory effect at low concentrations (100 mM and below), in multiple buffer systems, and in stark contrast to β-alanine, nonspecific inhibitory effects can be ruled out and the basis for the IRI activity of α-alanine can be addressed.

To investigate whether α-alanine has any effect on ice crystal morphology, and therefore binds ice, we used a modified version of the “sucrose-sandwich” assay. In this assay, concentrated sucrose solution (50% w/v) is used to produce segregated ice crystals whose individual shapes can be clearly observed. Crystals grown in the presence of α-alanine did not exhibit morphologies that are characteristic of ice-binding (Figure S2). This is reminiscent of other small molecule IRIs elsewhere discovered,^19^ in contrast to larger IRI-active materials (e.g., PVA, AFPs).^33−35^ In the standard “sucrose-sandwich” assay, we also observed no ice growth inhibition (Figure S3). We hypothesize that this is due to the high fraction of liquid (sucrose solution) in this assay, which means that diffusion effects are dominant and the effective concentration of amino acid at the ice/water interface is very low, resulting in no observable activity.

In the absence of ice shaping effects, we looked to identify an alternative mechanism to explain the IRI activities of α- and β-alanine that does not depend on ice binding. This amounts to a challenging task, given the transient nature of the ice/water interface during recrystallization and the size and similarities of these two structures. To overcome this, we used atomistic MD simulations to study the growth of ice in the presence of α- or β-alanine. These simulations comprise an ice/water interface featuring a central slab of ice and two adjacent slabs of water in contact with a vacuum, as depicted in Figure 2a. The ice slab is orientated so that a selected lattice face (Figure S4a) is exposed to the water in the *xy*-plane, seeding ice growth in the ±*z*-direction. We used the all-atomistic CHARMM36 force field^36^ along with the TIP4P/Ice model^37^ to simulate the amino acid and water molecules. Further details of the computational setup can be found in the Supporting Information. Using this setup, we collected 60 statistically independent trajectories for α-alanine as well as β-alanine: 20 for each of the prismatic and the basal faces. We also probed concentration effects by including either one or two molecules in each of the two water slabs in every trajectory. The simulations were run for 100 ns (prismatic faces) or 120 ns (basal face), by which time only a small quantity of liquid water typically remains in the simulation cell. We quantified ice growth by calculating the number of molecules in the seeded ice cluster over time, which was then converted to a growth rate. We note that while these simulations do not fully capture the complex recrystallization process (Ostwald ripening) in its entirety, the transfer of water molecules from the supercooled liquid fraction at the grain boundaries to the surface of a growing crystal is a fundamental step in ice recrystallization. Our simulations capture the kinetics of this process (i.e., the growth rate), which is thus directly proportional to the rate of ice recrystallization represented by the % MGS metric. This computational methodology has been validated extensively, and results obtained via this setup have shown excellent correlation with the experimentally observed IRI activity of both polymers^38^ and small peptides.^39^

**Figure 2:**
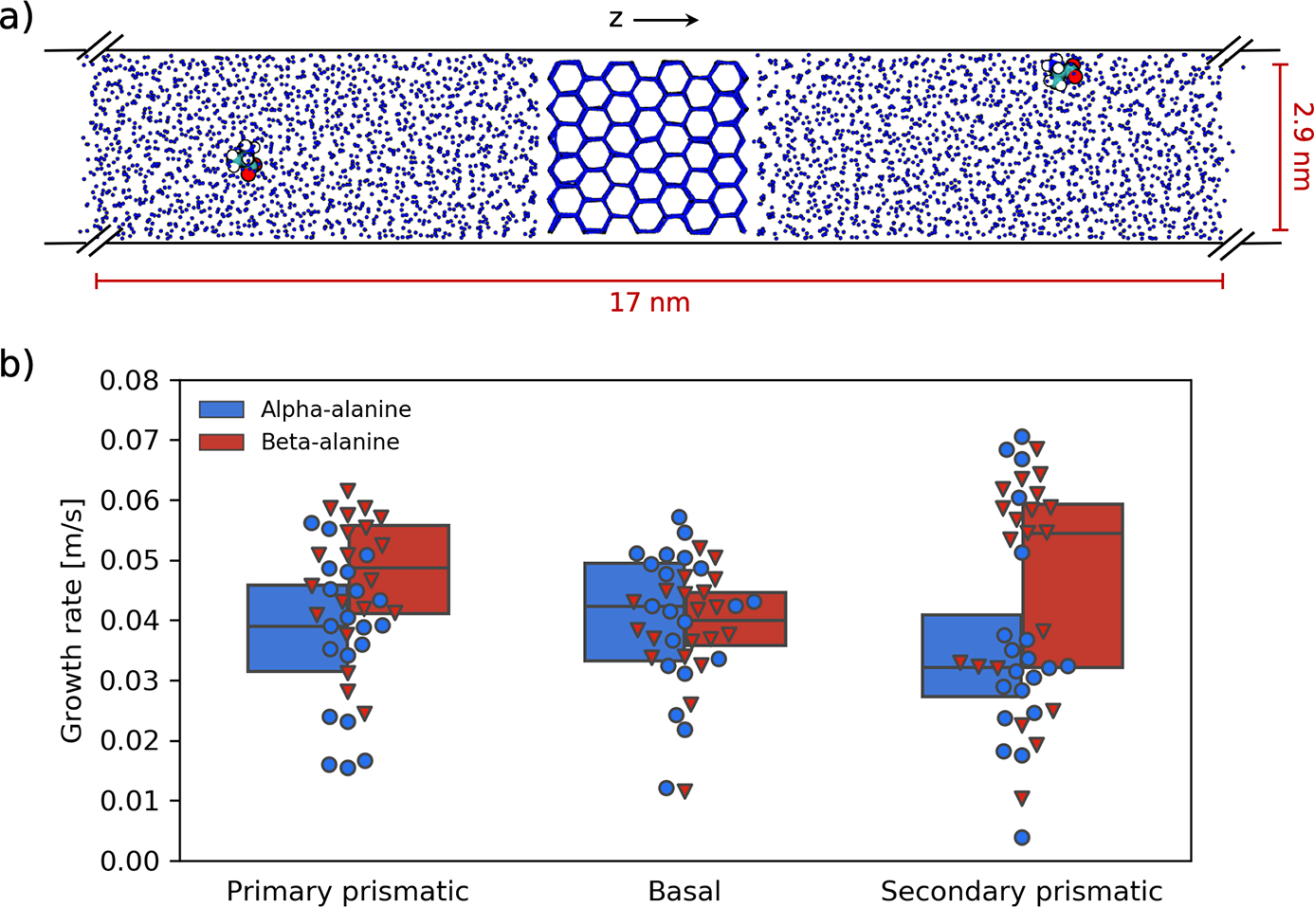
(a) Computational setup illustrating the growth of a primary prismatic plane in the ±*z* direction. (b) Rates of ice growth in simulations containing α- (left, circles) or β-alanine (right, triangles). The rate of ice growth is calculated over the period from when (α/β-)alanine first binds ice until the end of the simulation. Box plots in part b show the median and quartiles of the distribution.

In the simulations concerning the primary and secondary prismatic planes, we found that the presence of α-alanine resulted in slower rates of growth compared to β-alanine (Figure 2b), consistent with our experimental % MGS data. The basal fronts displayed similar rates of growth in the presence of either α- or β-alanine, although we note that in the context of IRI, growth (inhibition) at the prismatic fronts is considered of greater relevance due to the rapid growth rates observed from these faces relative to the basal face.^17,40^ To quantify these results categorically, we define a simulation as showing ice growth inhibition when the observed growth rate is below 0.03 m/s. This corresponds to a minimum of 50% reduction in the growth rate compared to our control simulations which contain no (α/β-)alanine molecules, although the following trends hold regardless of the chosen cutoff value within the range of 0.01 and 0.06 m/s. Applying this definition, we observed 5 and 6 instances of inhibition by α-alanine, and 1 and 2 instances by β-alanine, for the primary and secondary prismatic planes, respectively (Table 1).

**Table 1:** Outcomes of Simulations with Respect to (α/β-)Alanine Molecules Becoming Overgrown by Ice^a^

	α-alanine			β-alanine		
plane	OG^b^	IRI^c^	no IRI^d^	OG^b^	IRI^c^	no IRI^d^
primary prismatic	2 (4)	5 (8)	13 (8)	10 (13)	1 (3)	9 (4)
basal	4 (10)	3 (3)	13 (7)	7 (15)	1 (4)	12 (1)
secondary prismatic	7 (4)	6 (11)	7 (5)	15 (14)	2 (3)	3 (3)

a Numbers in parentheses show the outcomes for the simulations with two alanine molecules.

b A trajectory was defined as overgrown (OG) if at least one alanine molecule is deposited within the ice at least two layers (∼8 Å) deep along the *z*-axis with respect to the water by the end of the simulation.

c A trajectory was defined as showing ice growth inhibition (IRI) if the growth rate is below 0.03 m/s.

d The numbers for trajectories shown here do not include those which are overgrown (OG).

With our simulation and experimental data in agreement, we examined the trajectories looking for differences between α- and β-alanine that could explain their IRI activities. Interestingly, a significant number of (α/β-)alanine molecules were found to become overgrown by the advancing ice front and then incorporated into the lattice, as depicted in Figure S4b. We defined a trajectory as overgrown if at least one molecule of (α/β-)alanine is deposited under two or more layers (∼8 Å) of ice by the end of the simulation. These outcomes, summarized in Table 1, revealed that β-alanine is more frequently overgrown than α-alanine by each of the three crystal fronts studied. Again, we highlight these differences for the primary and secondary prismatic planes, where β-alanine is overgrown in 10 and 15 instances, respectively, compared to just 2 and 7 cases for α-alanine. Hence, this process readily occurs under these conditions, in contrast to larger molecules such as AFPs and polymers, for which overgrowth is considered a rare event at similar levels of supercooling.^41,42^

Having established this difference between α- and β-alanine, we suggest that inhibition and overgrown outcomes might be linked, because once the molecule becomes overgrown, the ice front can advance unimpeded and any inhibitory capacity is lost. In contrast, when the (α/β-)alanine molecule is not overgrown, it is still able to disrupt the growth of ice at or near the interface. Indeed, such differences can clearly be observed by comparing the growth rates of nonovergrown and overgrown trajectories (Figure S5). We also suggest that the overgrowth of (α/β-)alanine molecules has a compounding effect, as it sequesters the amino acids in the ice fraction. Consequently, the effective concentration of α-alanine at the grain boundaries could increase over time relative to β-alanine, further slowing ice growth via greater surface coverage. It is necessary to point out that there are cases wherein individual simulations, as with their experimental counterparts, do not reflect this overall trend. Fundamentally, IRI activity is not an “on/off” property, especially when examined at the scale of these simulations, and a large number of independent trajectories were therefore required to validate these findings. We also note that in the simulations where each ice front is exposed to two (α/β-)alanine molecules, the same trends in terms of ice growth inhibition and overgrowth are observed (Table 1, Figure S6). In these simulations, ice also grows at a consistently slower rate compared to those containing a single amino acid molecule (Figure S6), reflecting the concentration effects we observe experimentally (Figure 1a). Nonetheless, we focus our analysis herein on simulations with one (α/β-)alanine molecule, as we can clearly define these cases as overgrown (or not) with respect to a single molecule.

To understand why β-alanine is more likely to become overgrown compared to α-alanine, we sought to identify relevant characteristics that differ between these two molecules. For AFPs, the size of the molecule or area of its ice binding site is known to correlate strongly with antifreeze activity.^43,44^ Our previous work^38^ also revealed that, for the flexible polymer PVA, the effective volume and contact area with the ice surface can also determine the strength of IRI. Therefore, we first computed the volume and solvent-accessible surface area (SASA) occupied by these two molecules throughout the course of the simulations. However, we found that these properties of α-alanine and β-alanine are almost indistinguishable, with average volumes and surface areas differing by approximately 1 Å^3^ and 3 Å^2^ in volume and SASA, respectively, corresponding to a relative difference of around 1% for each quantity (Figure S7). Moreover, while α-alanine typically occupies a larger volume than β-alanine, this trend is reversed with respect to the surface areas. Hence, molecular volumes or surface areas do not appear to be correlated with IRI activity for these small molecules. We also investigated the (binding) orientation of the molecule with respect to the ice front but found no correlation between this property and IRI activity (data not shown).

Next, we analyzed the hydrogen bonding interactions between these molecules and water/ice. We found that the hydrogen bonding capacity of α- and β-alanine differ significantly, considering both molecules share the same hydrogen bond donor and acceptor groups. Whilst β-alanine frequently forms three or four hydrogen bonds with water via its carboxylate group, the ability of α-alanine to form a full complement of bonds via this same group is impaired (Figure S8, left panel). In fact, we observed that α-alanine typically forms just one or two hydrogen bonds out of a possible four. The number of hydrogen bonds formed via the amine group, meanwhile, does not appear to differ between these two compounds (Figure S8, right panel). This difference arises from the relative positions of the carboxylate and amine groups in these two molecules. For α-alanine, these groups are bound to the same (α-)carbon atom, whereas for β-alanine they are separated by an additional methylene group (Figure 1b). Consequently, the carboxylate and amine groups in α-alanine are fixed within close proximity, and the rotation of the carboxylate group (defined by the O–C–C–C/O–C–C–N dihedral angle for α- and β-alanine, respectively) is restricted due to the electrostatic interaction between the protonated nitrogen atom of the amine group and the nearest oxygen of the carboxylate moiety. We confirmed this using well-tempered metadynamics, employing the aforementioned dihedral angles as the collective variables. The resulting free energy landscape revealed two energy minima in this phase space for α-alanine, corresponding to two conformations wherein the distance between the nitrogen atom and each oxygen in turn is minimized (Figure S9a). The high energy barrier that exists between these conformers prohibits free rotation about the C– C bond, consistent with the narrow dihedral distribution observed in our unbiased simulations (Figure S9b). Hydrogen bond formation is sterically hindered in these conformations, limiting the hydrogen bond interactions between α-alanine and water. In contrast, the free energy landscape of β-alanine features conformational energy barriers on the same order as thermal fluctuations at room temperature, allowing this phase space to be fully explored during the unbiased run and greater hydrogen bond formation compared to α-alanine.

Given the difference in the hydrogen bonding capacities of α- and β-alanine, we investigated differences in the solvation shells of these two molecules. We determined the hydration index, based on the definition provided by Tam et al.,^19^ to be 0.152 ± 0.023 and 0.162 ± 0.021 molecules/Å^3^ for α- and β-alanine, respectively. This small difference suggests a marginally greater entropic gain associated with the desolvation of β-alanine compared to α-alanine. To consolidate these results, we also computed the solvation free energy, Δ*G*_solv_, for both species via MD simulations using the Bennett acceptance ratio method.^45^ The solvation energies of α- and β-alanine were found to be −36.9 ± 0.1 and −43.5 ± 0.2 kcal/mol, respectively, in line with solvation energies previously reported.^46^ The greater solvation energy of β-alanine compared to α-alanine is consistent with both hydrogen bonding and hydration index data. These results at first appear to counter intuition: β-alanine can form a greater number of hydrogen bonds with ice than α-alanine and stands to benefit from a larger entropic gain upon binding, yet it is less effective at inhibiting ice growth. We instead suggest that a stronger interaction with the ice front, confirmed by the aforementioned computational analyses, could be detrimental to the IRI activity of these small molecules as it increases the likelihood of becoming overgrown. Further, we also provide substantial evidence that the molecule’s compatibility within the ice lattice is crucial to the overgrowth outcome. To demonstrate this, we computed the distances between the atoms in (α/β-)alanine that are able to participate in hydrogen bonding (nitrogen and oxygen). We found that the nitrogen–oxygen distances provide a close match with the lattice distances of the prismatic and basal planes for β-alanine but not for α-alanine (Figure 3a). The lattice distances represent the oxygen–oxygen distances between water molecules in ice (Figure 3b), and therefore a closer match to these distances means that the molecule can be incorporated into a growing crystal at the lattice sites and overgrown without significant disruption to the crystal order.

**Figure 3:**
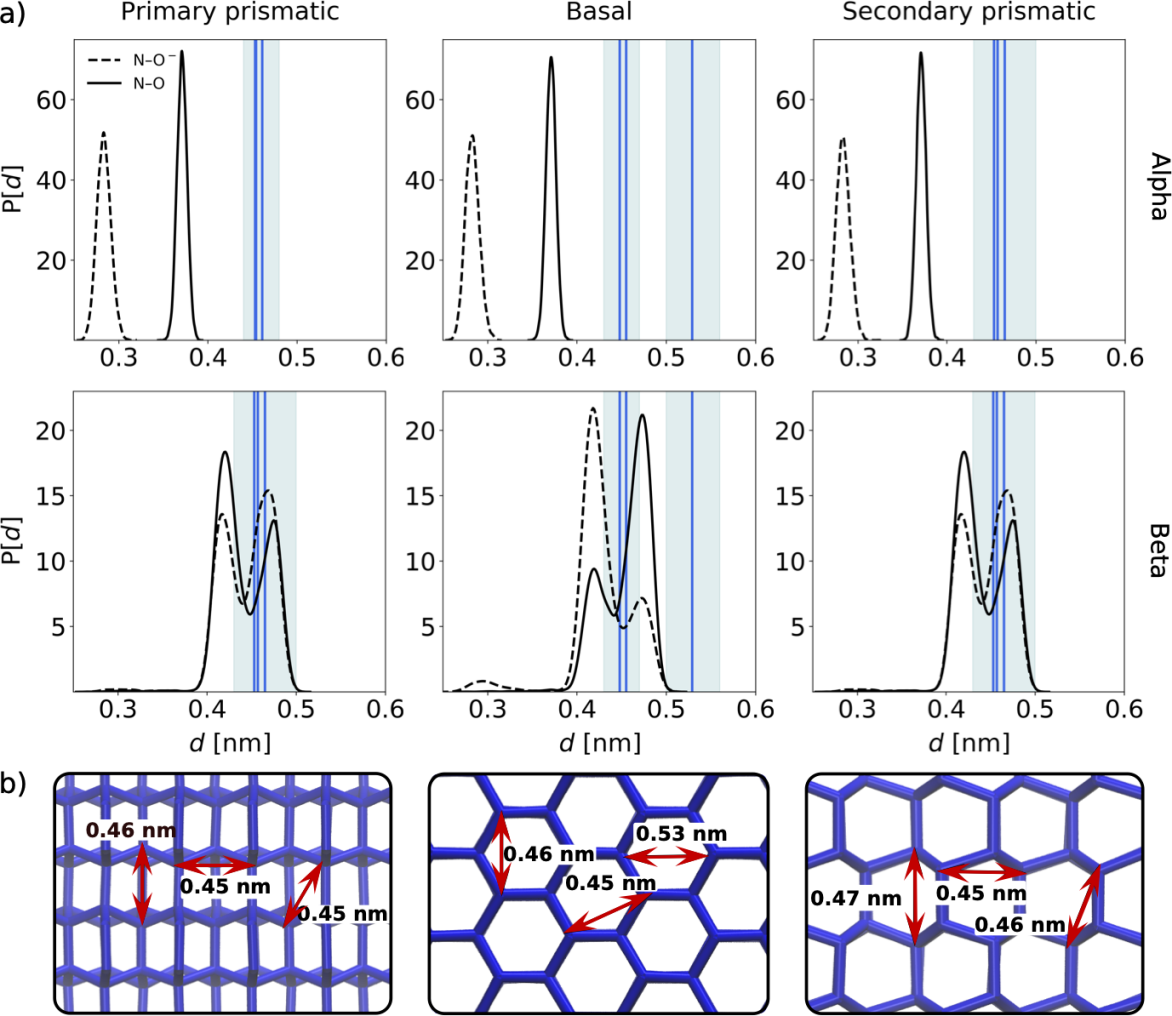
(a) (α/β-)alanine N–O distance distributions for all simulations. The solid blue lines represent the average ice lattice distance sampled from these trajectories. The shaded cyan area represents ±1 standard deviation. (b) Schematic showing the characteristic ice lattice distances for the primary prismatic, basal, and secondary prismatic faces (left to right). These faces are exposed to water in the *xy*-plane during the simulations.

Further, we also observed that β-alanine offers a more compatible “fit” within the crystal lattice than α-alanine with respect to its tetrahedral arrangement with neighboring water molecules. When a given water oxygen (O_W_) was replaced by an oxygen atom (O_Ala_) or nitrogen atom (N_Ala_) from β-alanine (Figure 4b), the corresponding angles between these atoms and water were in close agreement with the O···O···O angles observed in a tetrahedrally coordinated lattice structure consisting of just water molecules (Figure 4a and Figure S10). In contrast, the angles formed between the oxygen atoms of α-alanine showed a broader distribution and a greater deviation from the O···O···O angle in pure ice. Similarly, the geometry of (α/β-)alanine molecules that become overgrown (right panels, Figure 4a) also displayed greater tetrahedral character with coordinated water molecules than those that were not overgrown (left panels, Figure 4a). Hence, these data also provide rationale as to why certain molecules of either (α/β-)alanine become overgrown whereas others do not. Given that these small molecules have relatively few degrees of freedom with respect to their geometry, this highlights the subtlety of the features which can determine whether a molecule becomes overgrown and consequently the level of ice growth inhibition.

**Figure 4:**
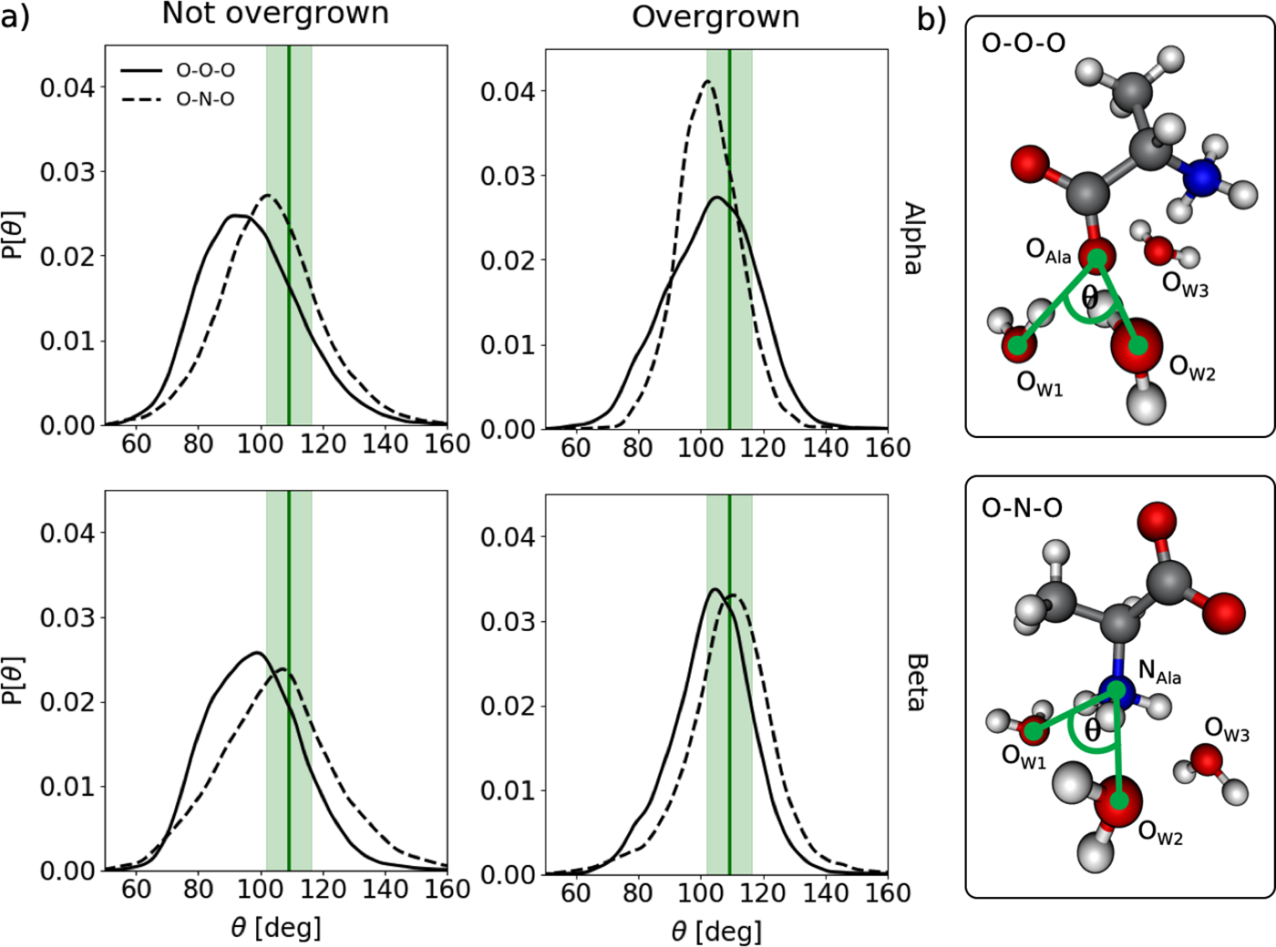
(a) O···O···O and O···N···O angle distributions for simulations of (α/β-)alanine with the primary prismatic plane of ice exposed. The solid green line represents the average O···O···O angle between tetrahedrally coordinated water molecules in the ice crystal, sampled from these trajectories. The shaded green area represents ±1 standard deviation of the sampled angles. These distributions are representative of the those observed for the basal and secondary prismatic simulations, which can be found in the Supporting Information (Figure S10). (b) Snapshot of α-alanine and water molecules showing representative O···O···O (top) and O···N···O (bottom) angles (θ). These angles are calculated for the three nearest water molecules (e.g., O_W1_–O_W3_) to (O_Ala_) (top) and (N_Ala_) (bottom), respectively, computed at every frame. For O···O···O angles, both O atoms of (α/β-)alanine are considered.

In summary, we have shown via α-alanine that very small molecules, with fewer than 15 atoms, can be effective IRI agents and represent a scaffold to understand structure-function relationships. We have also brought to attention amino acids as a new class of IRI-active materials, investigating the IRI activity of α-alanine alongside its isomer β-alanine, using quantitative experimental measurements and atomistic molecular simulations. Surprisingly, we found that the difference in IRI activity of these structures is dictated by their propensity to become engulfed and irreversibly overgrown by ice, underpinned by their compatibility to fit within the ice lattice. We note that the trends observed here with respect to ice binding significantly differ from those reported in the literature for, e.g., antifreeze proteins,^15^ highlighting the different structural determinants at play in small molecule IRIs. These findings provide new insights and avenues for the discovery and development of small molecule cryoprotectants, building upon the ubiquitous amino acid scaffold. In light of the limited IRI activity of α-alanine under high salt concentrations, the identification of saline-stable inhibitors represents a focal point for future work.

## Supporting Information Available

Additional experimental and computational details and methods including IRI and DIS assays and controls; MD and metadynamics simulations; geometry optimization and RESP charge fitting; determination of ice clusters and overgrowth; hydrogen bonding; calculation of molecular volumes, SASAs, hydration indices and solvation energies; supplementary figures including IRI controls; cryomicrographs from IRI and DIS assays; ice growth rates and data, volume, SASA and hydrogen bond distributions; free energy profiles; and O···O···O and O···N···O angle distributions (PDF)
Transparent Peer Review report available (PDF)
