# Correction to “Ice Recrystallization Inhibition by Amino Acids: The Curious Case of Alpha- and Beta-Alanine”

**DOI:** 10.1021/acs.jpclett.2c02044

**Published:** 2022-07-26

**Authors:** Matthew T. Warren, Iain Galpin, Fabienne Bachtiger, Matthew I. Gibson, Gabriele C. Sosso

In Figure 3a of the original Letter, the plot for β-alanine concerning the simulations of the primary prismatic plane (bottom left panel) is a duplicate of the plot for β-alanine concerning the secondary prismatic plane (bottom right panel). The corrected figure is provided here. The discussion pertaining to these results as well as the conclusions in the original Letter remain unchanged.

**Figure 3:**
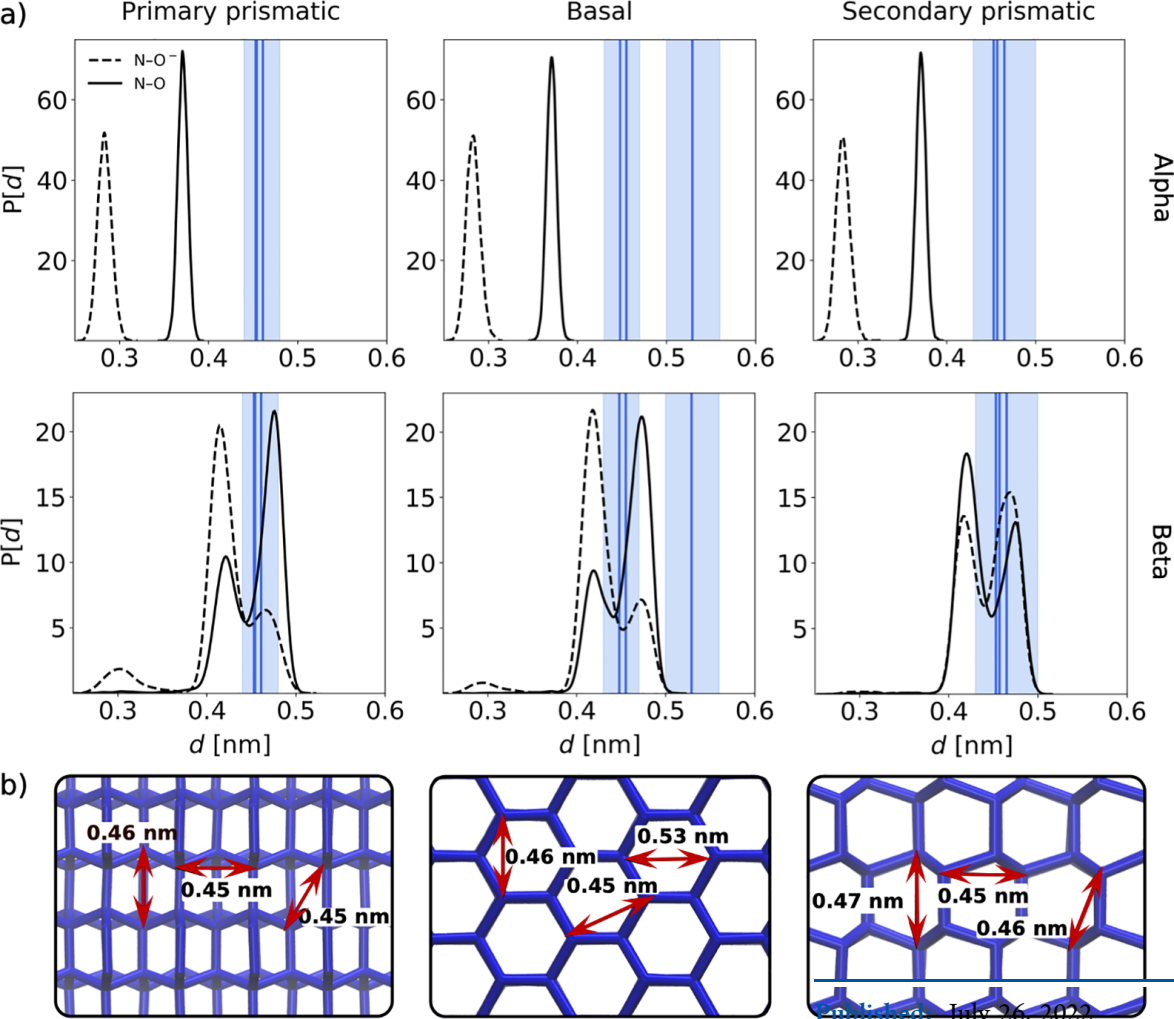
(a) (α/β-)Alanine N–O distance distributions for all simulations. The solid blue lines represent the average ice lattice distance sampled from these trajectories. The shaded cyan area represents ±1 standard deviation. (b) Schematic showing the characteristic ice lattice distances for the primary prismatic, basal, and secondary prismatic faces (left to right). These faces are exposed to water in the *xy*-plane during the simulations.

