# Supplementary material for: Ice Recrystallization Inhibition by Amino Acids: The Curious Case of Alpha- and Beta-Alanine
Source: J Phys Chem Lett. 2022 Mar 3;13(9):2237–44. doi: 10.1021/acs.jpclett.1c04080 (PMC9007522; doi:10.1021/acs.jpclett.1c04080)
Supplement: Supplementary file 1 — jz1c04080_si_001.pdf [file jz1c04080_si_001.pdf]

Ice Recrystallisation Inhibition by Amino Acids: The Curious Case of Alpha- and Beta-alanine SUPPORTING INFORMATION

Matthew T. Warren,[†,‡] Iain Galpin,[†] Fabienne Bachtiger,[†] Matthew I. Gibson,[†,‡] and Gabriele C. Sosso[*,†]

†*Department of Chemistry, University of Warwick, Gibbet Hill Road, Coventry CV4 7AL, United Kingdom*

‡*Warwick Medical School, University of Warwick, Gibbet Hill Road, Coventry CV4 7AL, United Kingdom*

E-mail: g.sosso@warwick.ac.uk

Supplementary Information

Experimental Methods

L-α-alanine, β-alanine and betaine were purchased from Sigma Aldrich. NaCl and PBS were purchased from Fischer Scientific. PBS was used at a 1X stock concentration, containing 137 mM NaCl, 10 mM phosphate and 2.7 mM KCl. HEPES buffer was also used at a 1X stock concentration, containing 140 mM NaCl, 50 mM HEPES and 1.5 mM phosphate. Tris-HCl was used at a 1 M concentration. 10 mM phosphate buffer contains 5.8 mM Na_2HPO_4 and 4.2 mM KH_2PO_4. All solutions and buffers were made in MilliQ water.

Splat cooling assay

Ice recrystallisation (inhibition) was measured using the "splat" cooling assay as previously described by Knight et al.[1] A 10 µL drop of each solution was dropped from a height of 1.4 m onto a glass coverslip placed on a thin aluminium plate cooled to −78 °C on dry ice. Upon impact with the coverslip, a polycrystalline ice wafer with an approximate diameter of 10 mm and thickness of 10 µm is formed instantly. The coverslip was then transferred to a Linkam Cryostage BCS196 pre-cooled to −8 ° C and left for 30 minutes at −8 ° C to anneal. Photographs were taken after 30 minutes via a Canon DSLR 500D digital camera using an Olympus CX 41 microscope equipped with a UIS-2 20x/0.45/∞/0-2/FN22 lens and crossed polarisers. The number of crystals in the field of view (FOV) were then counted using ImageJ,[2] and this number was divided by the FOV area to give the mean grain size (MGS). The MGS for each sample was then compared to the average MGS for the buffer solution only (i.e. a positive control for ice growth, unless otherwise stated), giving a percentage MGS relative to the control (% MGS). Each experiment was performed in triplicate, and % MGS values were reported as the mean across the three repeats.

IRI activity controls

As discussed in the main text, it was crucial to evaluate the solution conditions for IRI. It is essential to include saline (or other additives) in the solution to ensure a eutectic phase is formed, allowing for ice recrystallisation to occur. Typically, using pure water and low concentrations of solutes, no ice growth occurs, generating a false positive. Considering this, a buffer such as phosphate buffered saline (PBS) is typically employed (with ∼140 mM NaCl). However, this concentration of saline is not essential and relevant IRI data has been obtained in lower saline concentrations when using materials which are not stable or soluble in PBS.[3,4]

Figure S1 shows MGS data for 100 mM α-alanine, β-alanine, or betaine under different conditions. In PBS, there was no activity (ice growth) and in HEPES/TRIS buffers there

was also little evidence for IRI. 100 mM alanine in H_2O did inhibit ice growth, however to ensure this was not a false positive, betaine (IRI inactive small molecule) was also tested and in H_2O ice growth was observed. We hypothesise that the high molar concentrations of these small molecules (compared to what is used, for example, with proteins) ensures the eutectic phase still forms. Further study showed that 10 mM NaCl was tolerated by the system (IRI activity seen) and hence was used for the data shown in Figure 1. Similarly, α-alanine is IRI-active in 10 mM phosphate buffer, whereas β-alanine shows no activity under these conditions.

Dynamic ice shaping

Ice shaping was examined using a modified sucrose sandwich assay. Briefly, 2 µL of sample containing α- or β-alanine (or not in the case of the control) in 50% (w/v) sucrose solution were sandwiched between two circular glass coverslips each 14 mm in diameter. The coverslips were sealed using immersion oil to create a thin liquid film about 10 µm thick. The sample was cooled to -50 °C using a Linkam Cyrostage BCS196 to form a film of polycrystalline ice, and the temperature was then increased to -8 °C and held for 1 hour to anneal. The sample was subsequently heated at a rate of 0.2 °C/min until only 10-15 crystals remained and then cooled again at 0.1 °C/min during which the shapes of the ice crystals were observed. Images were captured at every 0.2 °C interval using an Olympus CX41 microscope equipped with a Canon DLSR 500D camera and a UIS-2 20x/0.45/∞/0-2/FN22 lens. Image processing was performed using ImageJ.[2]

Sucrose sandwich assay

"Sucrose-sandwich" assays were performed as described by Smallwood et al.[5] to measure ice recrystallisation inhibition. 2 µL of solution in 50% (w/v) sucrose were sandwiched between two 14 mm circular glass cover slips sealed at the edges using immersion oil and pressed together to form a liquid film around 10 µm thick. The "sandwich" was then transferred

to a Linkam Cryostage BCS196 and cooled to −50 °C at rate of 20 °C/min to induce the formation of polycrystalline ice. The samples were subsequently heated to −8 °C at a rate of 10 °C/min and thereafter held at −8 °C for 30 minutes during which images were taken every 5 minutes using a Canon DSLR 500D digital camera attached to an Olympus CX41 microscope equipped with a UIS-2 20x/0.45/∞/0-2/FN22 lens. Images were processed using ImageJ.[2]

Computational Methods

Molecular dynamics simulations

Molecular dynamics simulations were performed using the all-atomistic CHARMM36 forcefield[6] along with the TIP4P/Ice water model,[7] using the MD package GROMACS 5.1.3.[8] The simulations investigate the zwitterionic forms of α- and β-alanine and their impact on ice growth kinetics when exposed to an advancing ice front in either the primary prismatic, secondary prismatic or basal plane. Figure 2a provides an overview of the system setup where α-alanine is exposed to a growing primary prismatic plane. We ran 20 independent simulations of α- or β-alanine exposed to each of the three different ice planes (Figure S1b). Simulations involving the primary and secondary prismatic plane were run for 100 ns, whereas those involving the basal plane were run for 120 ns to account for slower growth kinetics of this plane.

The (α/β-)alanine structures were created using the Maestro2019[9] software package. Their geometries were optimised using the steepest-descent algorithm[10] along with their charge distribution using the restrained electrostatic potential (RESP) method[11] via the CP2K software package[12] (see below for more details). α- and β-alanine were then separately solvated in water within a cubic simulation box (edge = 3.4 nm) and equilibrated for 500 ps at room temperature and ambient pressure within the NPT ensemble: for this we used the Bussi-Donadio-Parrinello thermostat[13] and the Berendsen barostat,[14] with coupling constants of 0.5 and 4 ps, respectively. Periodic boundary conditions (PBCs) were applied

in x-, y- and z-dimensions and the integration time-step for the leap-frog algorithm was set to 2 fs. In parallel, a separate ice/water box was created using the Genice v.2.1.1[15] software package, starting by preparing an ice crystal (of dimensions: 2.7 x 2.9 x 3.0 nm, 3.1 x 2.7 x 2.7 nm and 3.1 nm x 2.9 nm x 2.6 nm for primary prismatic, basal, and secondary prismatic planes, respectively) ensuring that the respective ice faces were lying in the xy-plane of the simulation box: this then ensured that the crystal acted as the seed for the ice phase to grow along the $\pm$ z-direction (Figure 2a and Figure S1b).

The oxygen atoms within the ice slab were subjected to position restraints via a harmonic potential characterised by a spring constant of 1000 kJ/mol. The system was solvated, with two layers of water (each one ~7 nm thick) added below and above the ice seed totalling 20375 atoms. The simulation box was elongated along the z-direction to include ~10.5 nm of vacuum: at this point we switched to 2D (xy) PBCs in conjunction with 9-3 Lennard-Jones "walls"[16] positioned at the top and bottom of the box. This allowed us to use the Yeh and Berkowitz correction term[17] to the Ewald summation which further mitigates any potential artefacts due to the treatment of electrostatic interactions when dealing with a slab geometry.[18] The cutoff for the van der Waals and electrostatic interactions was set to 12 Å and 10 Å, respectively, and a switching function was used to bring the van der Waals interaction to zero at 12 Å. The geometry of the water molecules was constrained using the SETTLE algorithm[19] while the P-LINCS algorithm[20] was used to constrain the hydrogen bonds for α- and β-alanine at their equilibrium value. This setup has been extensively validated in some of our previous works, see e.g. Ref.[21] Next, the ice/water system was equilibrated (NPT ensemble, using a semi-isotropic pressure coupling scheme) at 300 K for 30 ns. Once the α- and β-alanine and ice/water systems were independently equilibrated, (α/β-)alanine was placed into the water layer (in both the $\pm z$ direction) of the ice/water system, as per Figure 2a. The system was then further equilibrated for 20 ns at 300 K and then cooled down to 265 K within 10 ns: (α/β-)alanine molecules were not restrained meaning they were free to diffuse through the entire water slab during equilibration. A

100/120 ns production run at 265 K followed, switching to the Parrinello-Rahman barostat[22] with a coupling constant of 4 ps. As the starting configurations of (α/β-)alanine in the water layer depended on the last frame in the equilibration run, all trajectories had independent starting configurations.

Geometry optimisation, RESP charge fitting and force field optimisation

DFT calculations were performed using the CP2K software package,[12] which is based on a mixed Gaussian and plane waves (GPW) approach. We employed Goedecker-type pseudopotentials[23] with four, one and six valence electrons for C, H and O respectively. The Kohn-Sham orbitals were expanded in a double zeta valence plus polarization (DZVP) Gaussian-type basis set. We have utilised the VdW-DF exchange-correlation (XC) functional[24] and a plane wave energy cutoff of 500 Ry. For all calculations we used a single cubic cell of edge equal to 20 Å. We considered five relaxed structures of α- and β-alanine which we obtained by means of short (∼10 ns) classical MD simulations. We have included in these five relaxed configurations around ten water molecules surrounding the (α/β-)alanine molecule, which are needed due to the instability of (α/β-)alanine zwitterions in the gas phase.[25,26] We first optimised the geometry of these structures using energy and force thresholds of 0.001 Bohr and 0.0001 Ha/Bohr, respectively, and then used the restrained electrostatic potential (RESP) method[11] to compute the partial charges on each atom. To this end we chose the parameters R_{min} and R_{max} to be 1.0 and 3.0, respectively, although we note that our results hold for a wide range of R_{min} and R_{max} values. These parameters define the radii of the spherical shell around each atom, enclosing the grid points for fitting. Once the charges were computed, we took the mean charge for each atom across the five conformations and compared them with those originally proposed by CHARMM general force field (CGenFF).[27] We found that the RESP charges were in close agreement (within ± 0.2 electron charge units) with CGenFF for α-alanine, but not for β-alanine. Thus, we have replaced the CGenFF charges with those obtained via the RESP method for β-alanine. Whilst we are aware that

this is only a qualitative improvement (as opposed to the actual re-parametrization of the CHARMM36 force field for β-alanine), we stress that our results hold irrespective of this change, which implies that our models are sufficiently accurate to capture the molecular interactions at the heart of the IRI activity discussed in this work.

Identifying icy molecules

In order to distinguish whether water belongs to the ice or liquid phase, the Steinhardt order parameters[28] were employed. First, we compute the 6-th order Steinhardt vectors $q_{6,m}(i)$ as:

$$q_{6,m}(i) = \frac{\sum_{j \neq i}^{N} \sigma(|\mathbf{r}_{ij}|) Y_{6,m}(\mathbf{r}_{ij})}{\sum_{j \neq i}^{N} \sigma(|\mathbf{r}_{ij}|)}, \qquad (1)$$

where $\mathbf{r}_{ij}$ is the distance vector between the ith and j^{th} atom, $Y_{6,m}$ is a spherical harmonic of order $\{6, m\}$ and σ is a switching function which determines the extent of the of coordination shell. Next, we combine the Steinhardt vectors and arrive at the following order parameter:

$$s_6(i) = \frac{\sum_{j \neq i}^{N} \sigma(|\mathbf{r}_{ij}|) \sum_{m=-6}^{6} q_{6,m}^{*}(i) \cdot q_{6,m}(j)}{\sum_{j \neq i}^{N} \sigma(|\mathbf{r}_{ij}|)} \qquad (2)$$

where the asterisk denotes complex conjugation. Using a clustering algorithm described in Ref.,[28] we identify the largest connected cluster of water molecules whereby oxygen atoms display a value of $s_6(i)$ greater than a certain threshold (0.45, see Ref.[28]). We therefore obtain the number of water molecules found within the largest ice cluster per trajectory frame, which invariably will be the seeded ice crystal that grows over time.

Determining whether alanine is overgrown in ice

Whilst visual inspection makes it clear which simulations show (α/β-)alanine overgrown by ice, we defined this quantitatively based on the following criteria: one or more alanine

molecules must be deposited under two or monolayers of ice (relative to the initial contact point) at the end of the simulation in order for the trajectory to be classified as overgrown. To determine this, we first calculated the z-coordinate of (α/β-)alanine atom closest to the seeded ice slab (i.e. the contact point) which we denoted Z_{Ala}. We then added (or subtracted, depending on whether the (α/β-)alanine lies in the water slab above or below the seeded ice slab) 0.79, 0.75 or 0.91 nm (i.e. the z-distance between two oxygen atoms in equivalent lattice sites) to the z-coordinate for primary prismatic, basal and secondary prismatic faces respectively, resulting in Z_{OG}. Then we calculated the z-coordinate of the icy oxygen (Z_{Ice}) in the system that is furthest from the seeded ice slab. If Z_{Ice} is greater than Z_{OG}, and the number of water molecules with z-coordinates greater than Z_{Ala} is greater than 192, 192 or 256 for primary prismatic, basal and secondary prismatic faces respectively (i.e. the number of molecules in a two monolayer slab of ice), we class this trajectory as overgrown.

Determining ice growth rate and inhibition

The ice growth rate was calculated by first determining the size of the largest (in terms of number of molecules) ice cluster at the end of the simulation (t). From this, we subtracted the number of molecules in the largest ice cluster when (α/β-)alanine first binds to ice via two or more hydrogen bonds (t_0), giving the number of molecules added to the cluster over a period of time. We then calculated the average number of molecules in two monolayers of ice and the corresponding length of these layers in the z-dimension, which corresponded to 192, 192 and 256 molecules, and 0.79, 0.75 and 0.91 nm for the primary prismatic, basal, and secondary prismatic planes, respectively. The number of molecules per layer and the average layer z-length were then used to determine the growth rate in m/s. Ice growth inhibition was defined using a growth rate cutoff of below 0.03 m/s, which equates to a minimum 50% reduction in growth rate compared to our control simulations which contained no inhibitor molecules.

Molecular volumes and surface areas

Molecular volumes and solvent accessible surface areas were calculated using RDKit[29] and FreeSASA[30] libraries respectively. These quantities were computed for 1000 structures per trajectory, sampled at regularly spaced intervals.

Identifying hydrogen bonds

Hydrogen bonds were defined using geometric criteria based on an acceptor-donor bond distance of less than 2.90 nm and a donor-hydrogen-acceptor angle between 165° and 200°.

Metadynamics simulations

Metadynamics simulations were performed using PLUMED,[31,32] version 2.4.2. For α-alanine we used the O−C−C−N torsion angle as the collective variable (CV) to bias, with Gaussian potentials of width (σ) 0.1 nm and height (W) 0.8 kJ/mol being deposited every 500 steps. For β-alanine the O−C−C−C torsion angle was used as the CV and the width, height and deposition stride of the potentials were 0.1 nm, 0.1 kJ/mol and 500, respectively.

Lattice compatibility

The O···O···O and O···N···O angles represent the angle formed between the oxygen (O_{Ala}) or nitrogen (N_{Ala}) atom of (α/β-)alanine, respectively, and the oxygen atoms (O_W) of two neighboring water molecules, as depicted in Figure 4b. These angles were calculated for up to three nearest waters within an $O/N_{Ala}-O_W$ cutoff distance of 0.3 nm, producing up to 6 angles per O/N_{Ala}. For O···O···O angles, angles were calculated for both O atoms of (α/β)-alanine.

Hydration indices

To calculate the hydration index for α- and β-alanine, each molecule was first equilibrated in a simulation cell containing around 3000 water molecules via a 15 ns NPT simulation at

300 K. The system was subsequently cooled to 265 K over 10 ns, followed by another 20 ns at 265 K. 500 frames obtained from the the final 10 ns of this run were then used for analysis. To determine the radius of the first solvation shell of (α/β-)alanine, the shortest distance between each water molecule (O_W atom) and the amino acid was first determined at each frame, up to a cutoff radius of 0.5 nm from (α/β-)alanine. These distances were binned at every frame, and a probability density distribution was obtained by normalising the sum of each bin by the sum of all the bins and the bin width. The resulting probability density function was then smoothed and the radius corresponding to the second local minimum of this function was determined (Figure S11). The number of water molecules within this distance from (α/β-)alanine, which we denote as the hydration number, was then determined for every frame, as was the molecular volume of (α/β-)alanine using the RDKit[29] package. A hydration index was then obtained for each frame by dividing the hydration number by the corresponding molecular volume.

Solvation energies

The solvation free energy ΔG_{solv} has been computed as: $\Delta G_{solv} = \Delta G_{coulomb} + \Delta G_{vdW}$, where $\Delta G_{coulomb}$ and ΔG_{vdW} correspond to the contributions of long-range electrostatic (Coulomb) interactions and van der Waals (vdW) interactions, respectively. The GROMACS package allows for the intramolecular interactions to persists as we decouple the intermolecular interactions, thus avoiding the need for an additional in vacuo calculation (to turn the intramolecular non-bonded interactions on again). The Bennett Acceptance Ratio (BAR) method[33] has been used to compute ΔG_{solv}. To this end, we equilibrated (α/β-)alanine in a simulation box containing 2932 water molecules via a 10 ns-long NPT simulation. The path from the fully interacting system (λ_0) to the de-coupled system (λ_1, where Coulomb and vdW interactions are both absent) was divided into 20 steps, whereby two coupling parameters $\lambda_{coulomb}$ and λ_{vdW} controlled the extent of Coulomb and vdW interactions, respectively. Firstly, vdW interactions were switched off, via increasing λ_{vdW} from 0

to 1 in 10 steps ($\Delta\lambda_{vdW} = 0.1$). Then, whilst keeping $\lambda_{vdW} = 1$, we switched off the Coulomb interactions by increasing $\lambda_{coulomb}$ from 0 to 1 in 10 steps ($\Delta\lambda_{coulomb} = 0.1$). We have verified that different paths in the $\lambda_{vdW}/\lambda_{coulomb}$ space (including the reverse path from λ_0 to λ_1) yield identical results.

Supplementary Figures

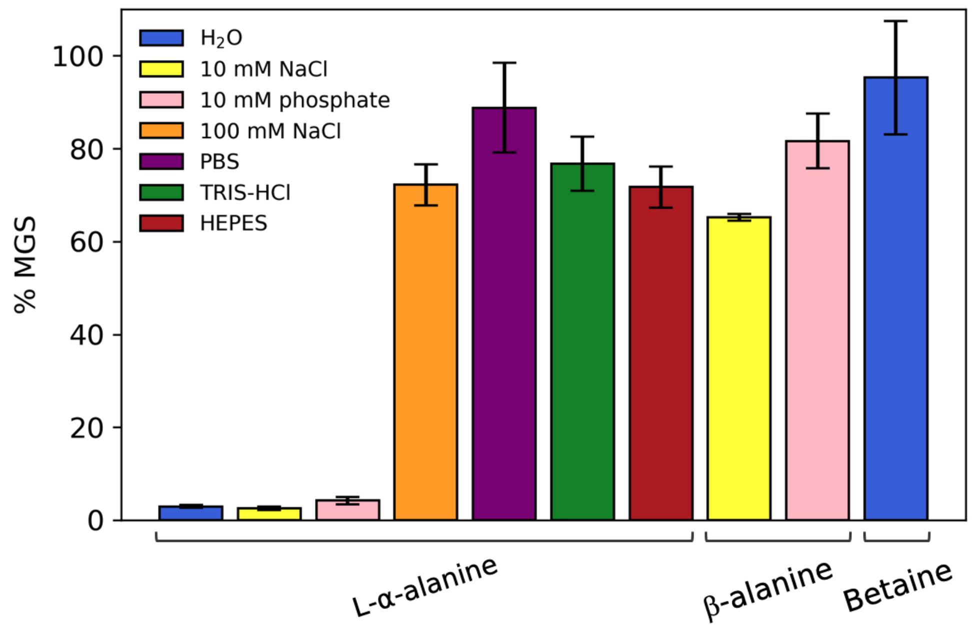

Figure 1: Ice recrystallisation inhibition activity of 100 mM L-α-alanine, β-alanine or betaine under different solution conditions. Each data point is reported relative to the MGS of the solution (without amino acid) alone, except in the case of pure water (as pure water does not ripen). A negative control of 100 mM L-α-proline in water (as this amino acid does not slow ice growth) was used in this case.

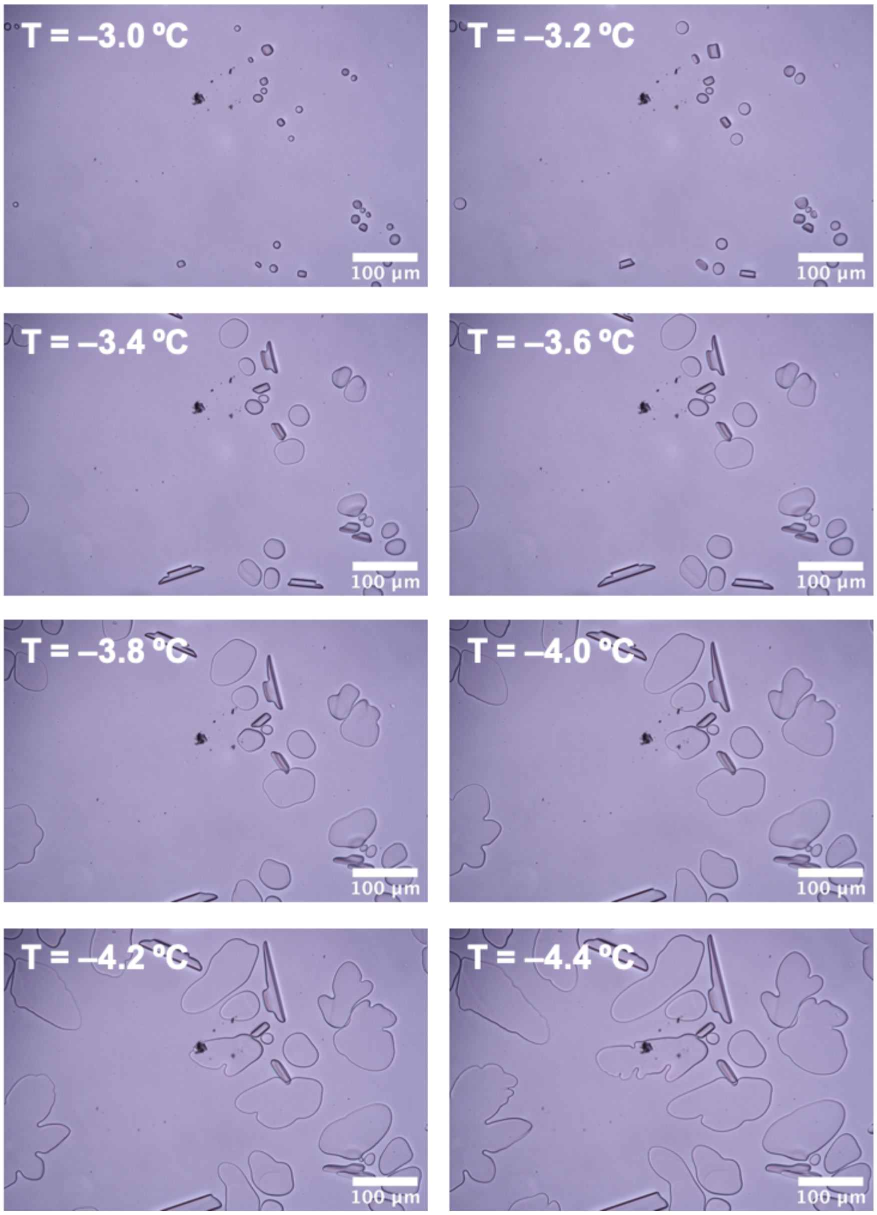

Figure 2: Cryomicrographs from modified sucrose sandwich assay for 200 mM L-α-alanine. Crystals which have needle-like shapes are flat disks on their sides with basal plane perpendicular to the viewing angle.

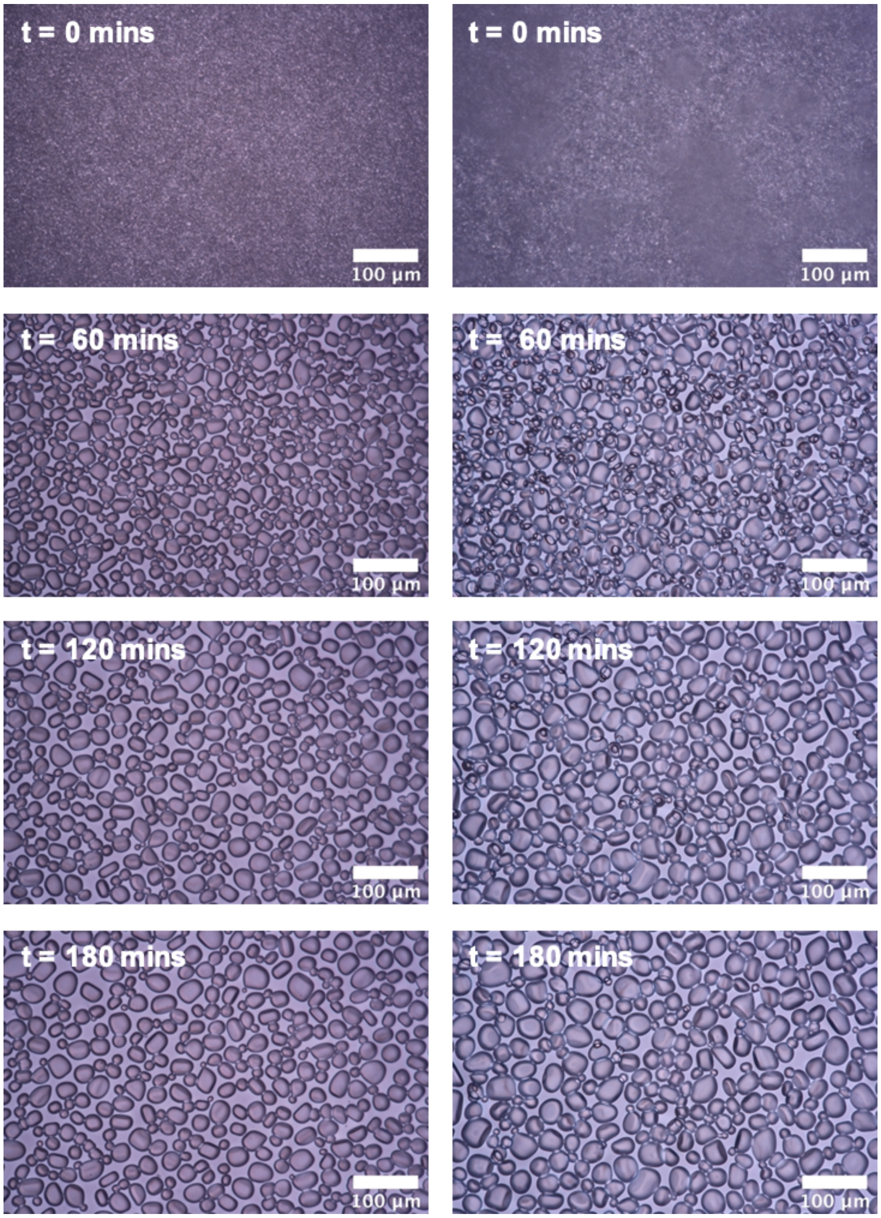

Figure 3: Cryomicrographs from sucrose sandwich assay for 200 mM L-α-alanine (right panels) compared to a positive control for ice growth (50 %wt sucrose only, left panels).

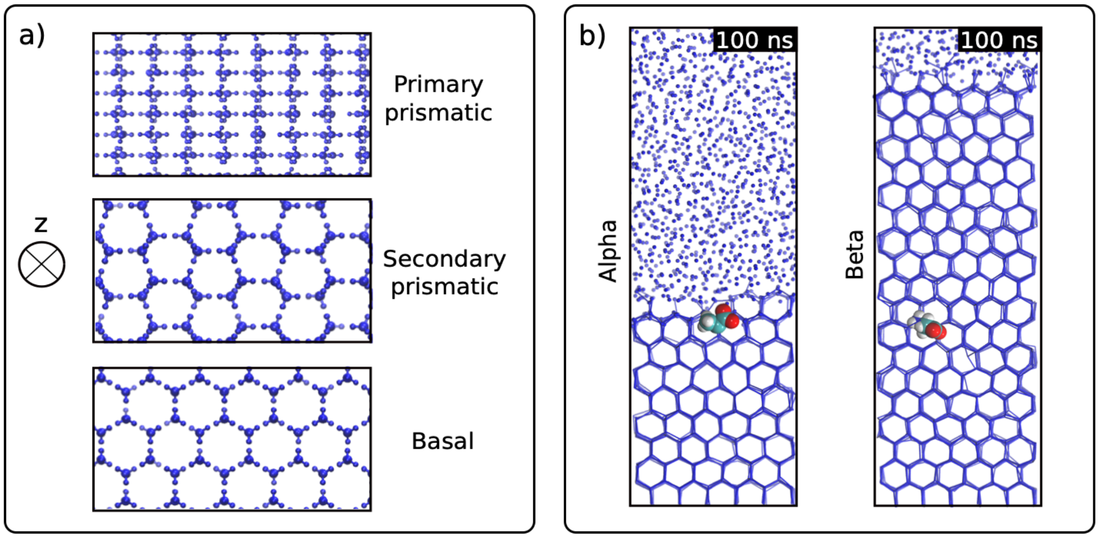

Figure 4: a) Snapshots of the different ice planes being investigated as viewed from the direction of ice growth (z-direction). b) Representative example of simulations of α- and β-alanine. Within the same time interval, β-alanine gets irreversibly overgrown in ice, losing any potential IRI activity.

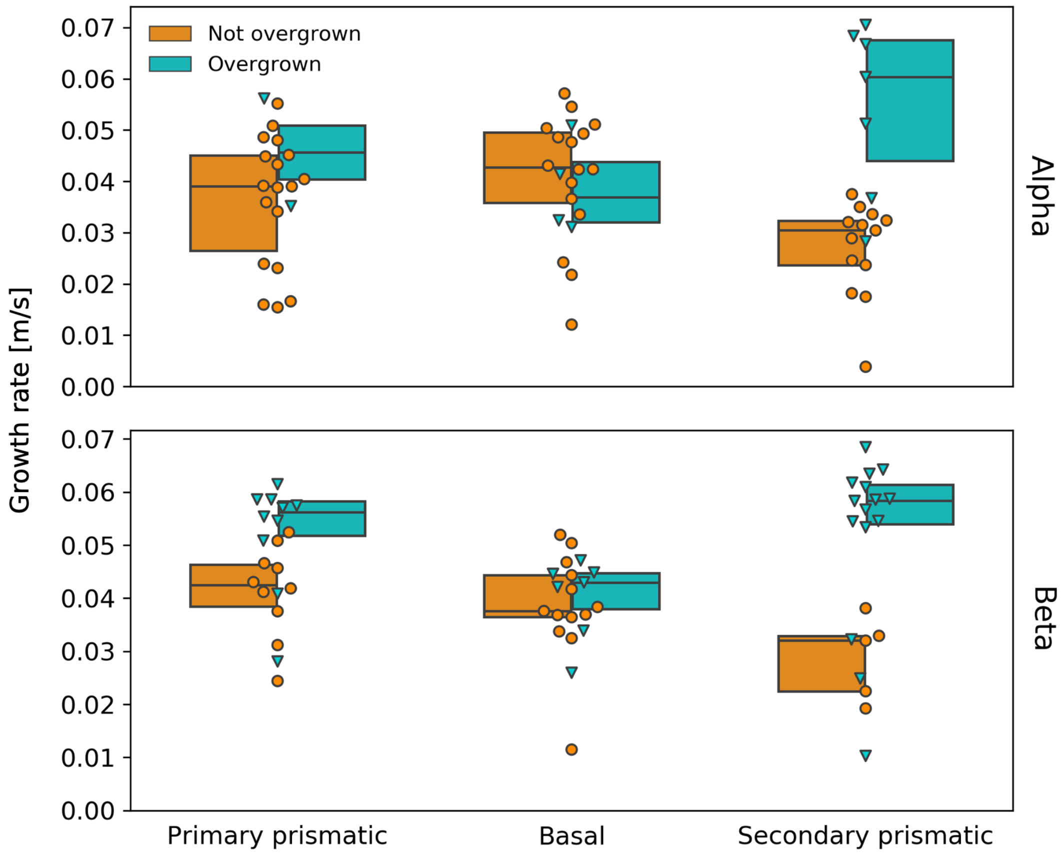

Figure 5: Rate of ice growth in simulations where (α/β-)alanine becomes overgrown (left, circles) compared to simulations where it is not overgrown (right, triangles). The rate of ice growth is calculated over the period from when (α/β-)alanine first binds ice until the end of the simulation. Whether a trajectory is classified as overgrown is defined as previously described. The boxes show the median and quartiles of the distribution.

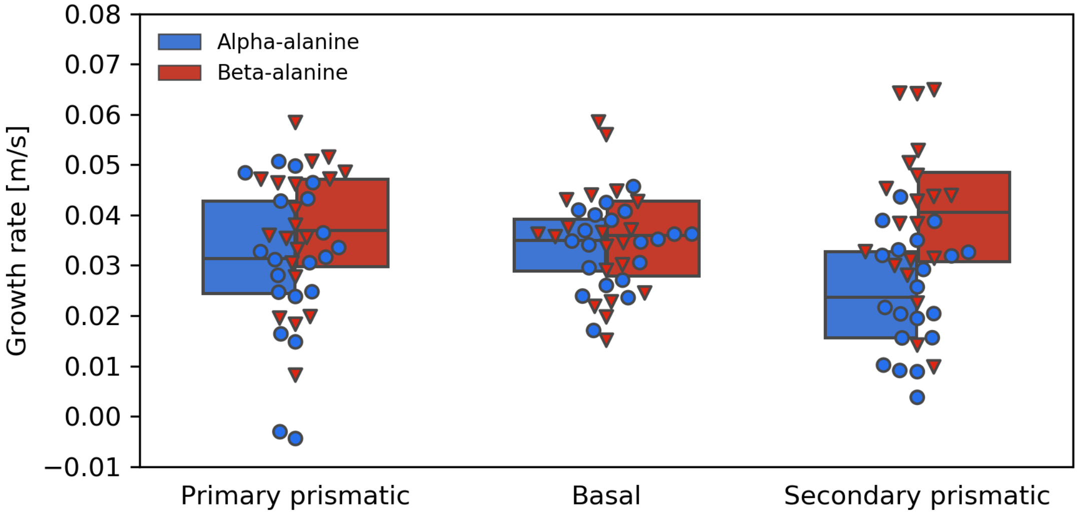

Figure 6: Rate of ice growth in simulations with two α-alanine (left, circles) or two β-alanine molecules (right, triangles). The rate of ice growth is calculated over the period from when (α/β-)alanine first binds ice until the end of the simulation. The boxes show the median and quartiles of the distribution.

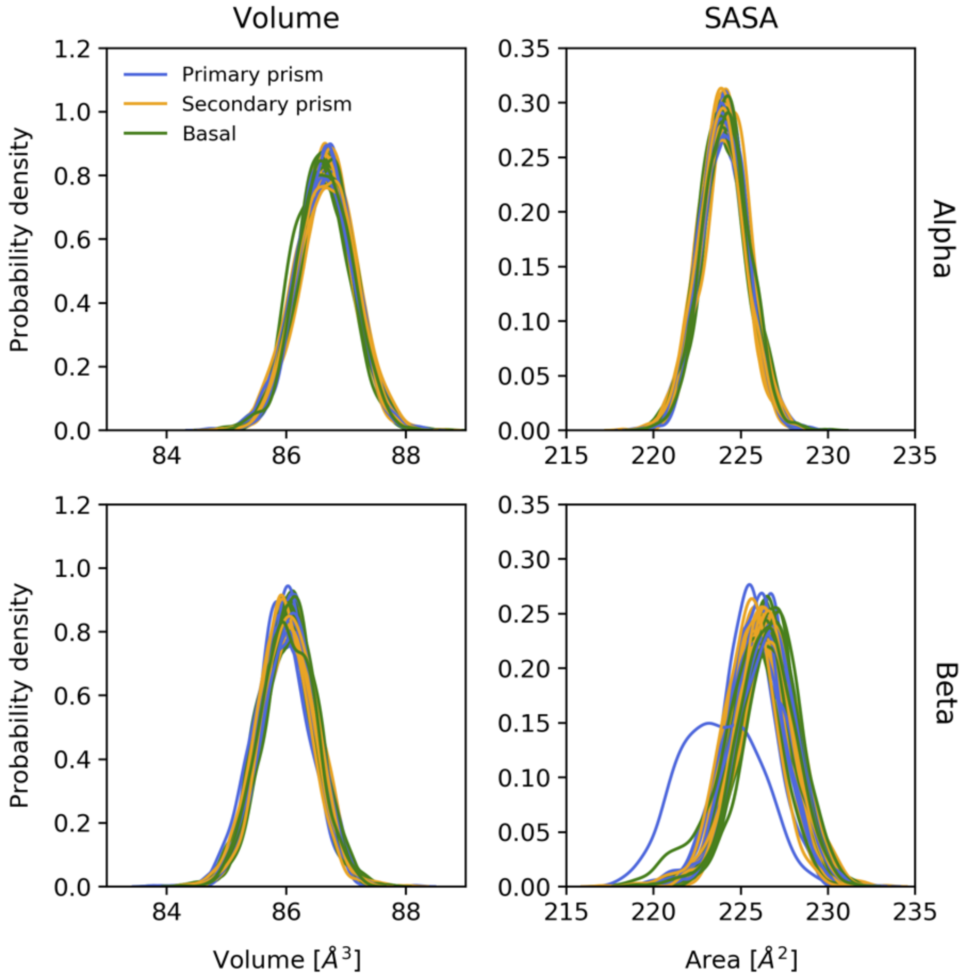

Figure 7: Distributions of volumes (left) and solvent accessible surface areas (SASA, right) for α-alanine (top) and β-alanine (bottom) molecules from molecular simulations. Each line represents the distribution for an independent trajectory.

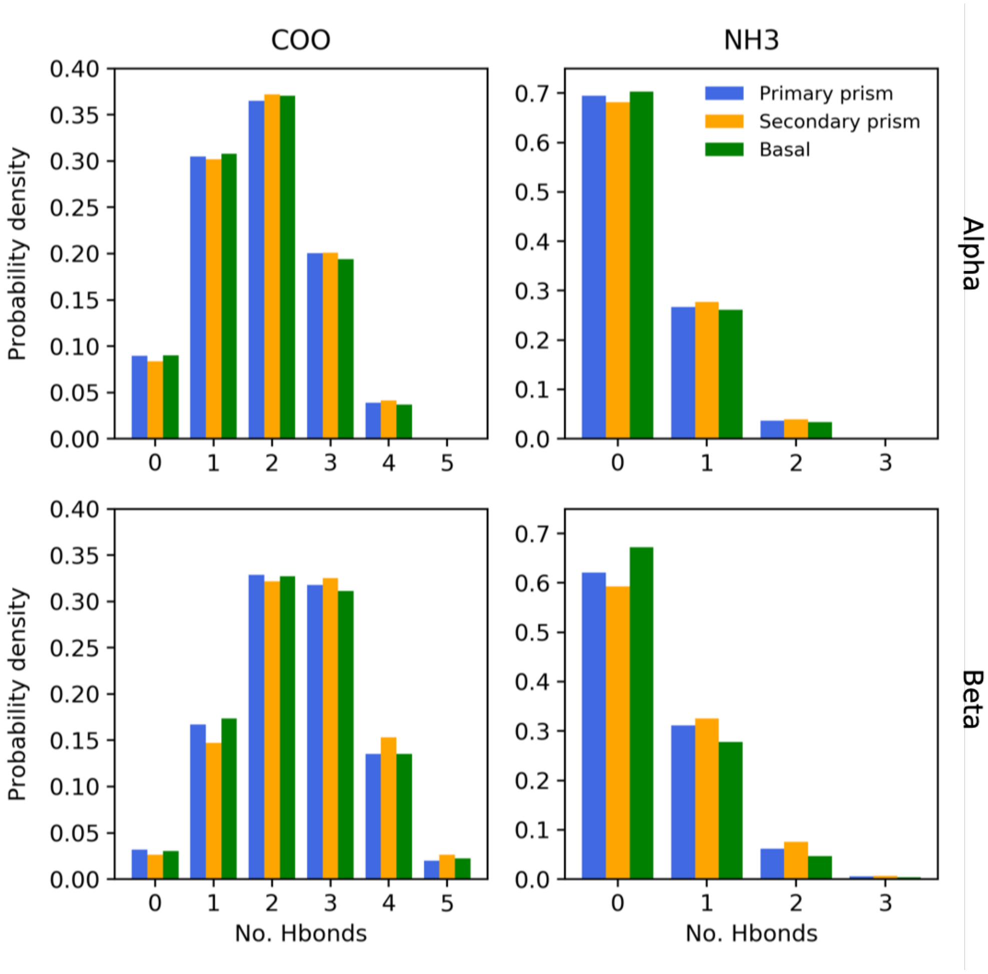

Figure 8: Distributions of hydrogen bonds formed between the carboxylate (COO^-) and amine (NH_3^+) groups of α-alanine (top) and β-alanine (bottom) with water or ice.

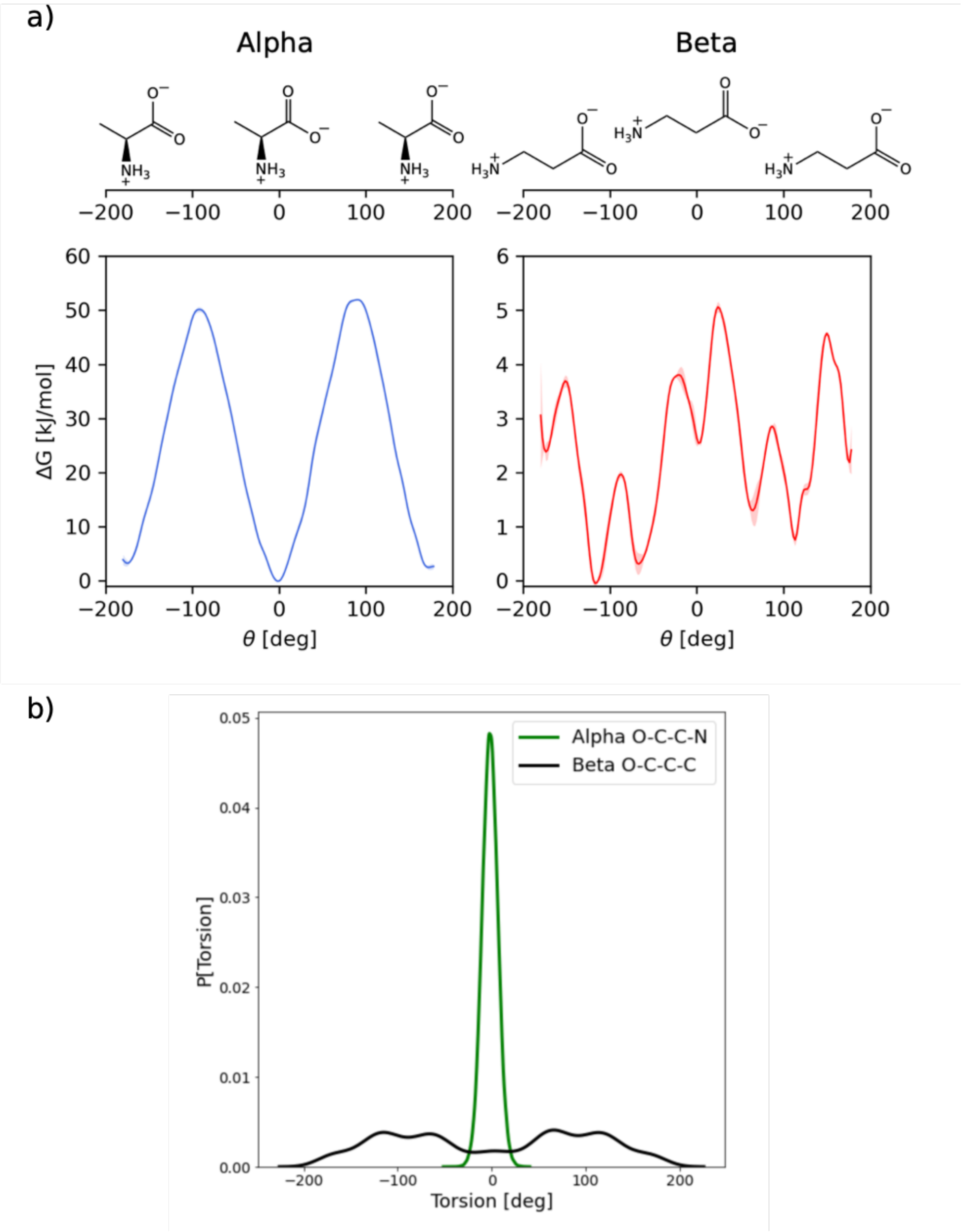

Figure 9: a) Conformational landscape of α-alanine and β-alanine. Gibbs free energy (ΔG) profile as a function of the O−C−C−N (α-alanine) and O−C−C−C (β-alanine) dihedral angle obtained via metadynamics simulations. The shaded region represents the standard error associated with the estimate of ΔG. Representative structures are shown above for dihedral angles of −180, 0 and 180 degrees (left to right). b) Dihedral angle distributions for α-alanine and β-alanine observed in the unbiased simulations.

.

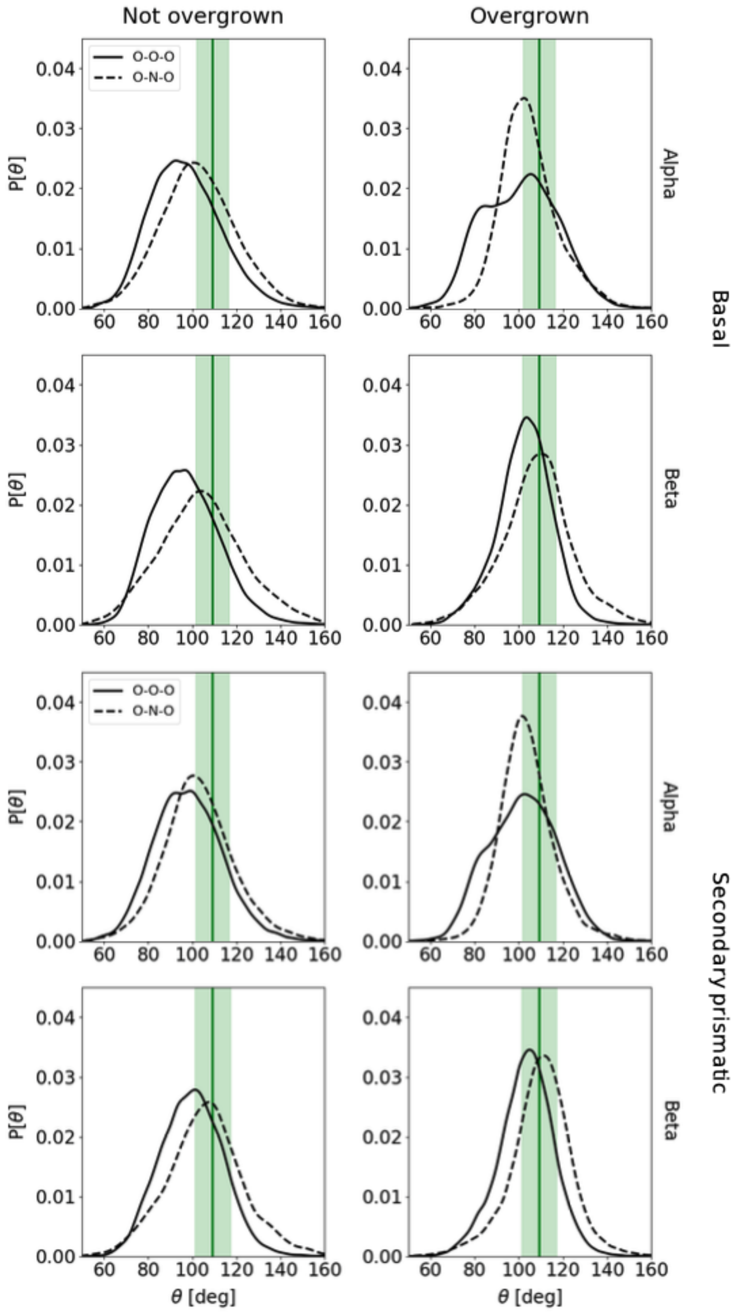

Figure 10: O···O···O and O···N···O angle distributions for simulations of (α/β)-alanine with the basal (top) and secondary prismatic (bottom) plane of ice exposed. Solid green line shows the average O···O···O lattice angle between neighboring water molecules in the ice crystal (θ) sampled from these trajectories with shaded green area showing ± standard deviation.

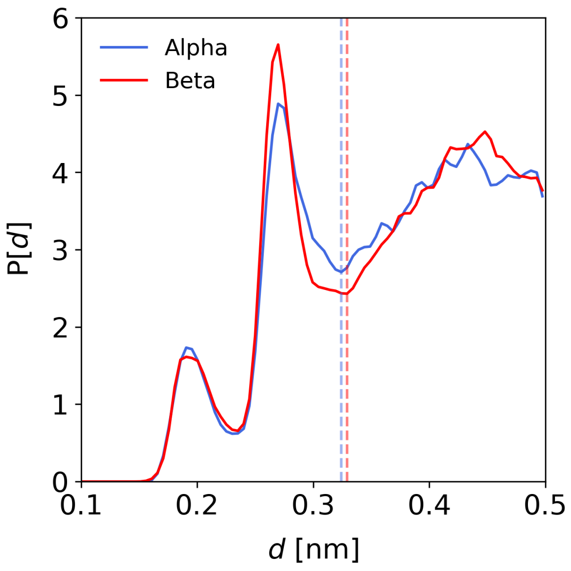

Figure 11: Smoothed probability density distributions of the distances between the O_W atoms of α-alanine (blue) or β-alanine (red) and water molecules, up to 0.5 nm from the amino acid. Dashed lines shows the distance corresponding to the first solvation shells of (α/β-)alanine, defined as the local minima. These distances represent the distance cutoffs used to determine the hydration numbers for (α/β-)alanine.

References

(1) Knight, C. A.; Hallett, J.; DeVries, A. L. Solute effects on ice recrystallization: An assessment technique. *Cryobiology* **1988**, *25*, 55–60.

(2) Rueden, C. T.; Schindelin, J.; Hiner, M. C.; DeZonia, B. E.; Walter, A. E.; Arena, E. T.; Eliceiri, K. W. ImageJ2: ImageJ for the next generation of scientific image data. *BMC bioinformatics* **2017**, *18*, 1–26.

(3) Georgiou, P. G.; Marton, H. L.; Baker, A. N.; Congdon, T. R.; Whale, T. F.; Gibson, M. I. Polymer Self-Assembly Induced Enhancement of Ice Recrystallization Inhibition. *Journal of the American Chemical Society* **2021**, *143*, 7449–7461.

(4) Balcerzak, A. K.; Febbraro, M.; Ben, R. N. The importance of hydrophobic moieties in ice recrystallization inhibitors. *RSC Advances* **2013**, *3*, 3232–3236.

(5) Smallwood, M.; Worrall, D.; Byass, L.; Elias, L.; Ashford, D.; Doucet, C. J.; Holt, C.; Telford, J.; Lillford, P.; Bowles, D. J. Isolation and characterization of a novel antifreeze protein from carrot (Daucus carota). *Biochemical Journal* **1999**, *340*, 385–391.

(6) Guvench, O.; Mallajosyula, S. S.; Raman, E. P.; Hatcher, E.; Vanommeslaeghe, K.; Foster, T. J.; Jamison, F. W.; Mackerell, A. D. CHARMM additive all-atom force field for carbohydrate derivatives and its utility in polysaccharide and carbohydrate- protein modeling. *Journal of Chemical Theory and Computation* **2011**, *7*, 3162–3180.

(7) Abascal, J. L. F.; Sanz, E.; Fernández, R. G.; Vega, C. A potential model for the study of ices and amorphous water : TIP4P / Ice. *The Journal of Chemical Physics* **2005**, *122*.

(8) Abraham, M. J.; Murtola, T.; Schulz, R.; Páll, S.; Smith, J. C.; Hess, B.; Lindah, E. Gromacs: High performance molecular simulations through multi-level parallelism from laptops to supercomputers. *SoftwareX* **2015**, *1-2*, 19–25.

(9) Schroedinger Release 2019, Maestro, Schroedinger, LLC, New York.

(10) Mayne, D. Q. A Stochastic Steepest-Descent Algorithm. *Journal of optimization theory and applications* **1988**, *59*, 307–323.

(11) Bayly, C. I.; Cieplak, P.; Cornell, W. D.; Kollman, P. A. A well-behaved electrostatic potential based method using charge restraints for deriving atomic charges: The RESP model. *Journal of Physical Chemistry* **1993**, *97*, 10269–10280.

(12) Kühne, T.; et al, CP2K: An electronic structure and molecular dynamics software package - Quickstep: Efficient and accurate electronic structure calculations. *Journal of Chemical Physics* **2020**, *152*, 194103–194147.

(13) Bussi, G.; Donadio, D.; Parrinello, M. Canonical sampling through velocity rescaling. *Journal of Chemical Physics* **2007**, *126*, 1–7.

(14) Berendsen, H. J. C.; Postma, J. P. M.; van Gunsteren, W. F.; DiNola, A.; Haak, J. R. Molecular dynamics with coupling to an external bath. *Journal of Chemical Physics* **1984**, *81*, 3684–3690.

(15) Matsumoto, M.; Yagasaki, T.; Tanaka, H. GenIce: Hydrogen-Disordered Ice Generator. *Journal of Computational Chemistry* **2017**, *39*, 61–64.

(16) Abraham, F. F.; Singh, Y. The structure of a hard-sphere fluid in contact with a soft repulsive wall. *The Journal of Chemical Physics* **1977**, *67*, 2384–2385.

(17) Yeh, I.-C.; Berkowitz, M. Ewald summation for systems with slab geometry. *Journal of Chemical Physics* **1999**, *111*, 3155–3162.

(18) Bostick, D.; Berkowitz, M. L. The Implementation of Slab Geometry for Membrane-Channel Molecular Dynamics Simulations. *Biophysical Journal* **2003**, *85*, 97–107.

(19) Miyamoto, S.; Kollman, P. A. Settle: An analytical version of the SHAKE and RATTLE algorithm for rigid water models. *Journal of Computational Chemistry* **1992**, *13*, 952–962.

(20) Hess, B. P-LINCS: A Parallel Linear Constraint Solver for Molecular Simulation. *J Chem Theory Comput.* **2008**, *4*, 116–22.

(21) Sosso, G. C.; Whale, T. F.; Holden, A. M.; Pedevilla, P.; Murray, B. J.; Michaelides, A. Unravelling the Origins of Ice Nucleation on Organic Crystals. *Chemical Science* **2018**, *9*, 8077–8088.

(22) Parrinello, M.; Rhaman, A. Polymorphic transitions in single crystals : A new molecular dynamics method. *Journal of Applied Physics* **1981**, *52*, 7182–7190.

(23) Goedecker, S.; Teter, M. Separable dual-space Gaussian pseudopotentials. *Physical Review B* **1996**, *54*, 1703–1710.

(24) Dion, M.; Rydberg, H.; Schröder, E.; Langreth, D. C.; Lundqvist, B. I. Van der Waals density functional for general geometries. *Physical Review Letters* **2004**, *92*, 246401.

(25) Ojha, A. K.; Vyas, N.; Dubey, S. P. Gas phase structural stability of neutral and zwitterionic forms of alanine in presence of (H2O)n=1–7 clusters: A density functional theory study. *Computational and Theoretical Chemistry* **2012**, *1002*, 16–23.

(26) Ghassemizadeh, R.; Moore, B.; Momose, T.; Walter, M. Stability and IR Spectroscopy of Zwitterionic Form of β-Alanine in Water Clusters. *Journal of Physical Chemistry B* **2019**, *123*, 4392–4399.

(27) Vanommeslaeghe, K.; Hatcher, E.; Acharya, C.; Kundu, S.; Zhong, S.; Shim, J.; Darian, E.; Guvench, O.; Lopes, P.; Vorobyov, I.; Mackerell, A. D. CHARMM general force field: A force field for drug-like molecules compatible with the CHARMM all-

atom additive biological force fields. *Journal of Computational Chemistry* **2010**, *31*, 671–690.

(28) Tribello, G. A.; Giberti, F.; Sosso, G. C.; Salvalaglio, M.; Parrinello, M. Analyzing and Driving Cluster Formation in Atomistic Simulations. *Journal of Chemical Theory and Computation* **2017**, *13*, 1317–1327.

(29) Landrum, G. RDKit: Open-Source Cheminformatics Software. **2016**,

(30) Mitternacht, S. FreeSASA: An open source C library for solvent accessible surface area calculations. *F1000Research* **2016**, *5*.

(31) Bonomi, M. et al. Promoting transparency and reproducibility in enhanced molecular simulations. 2019.

(32) Tribello, G. A.; Bonomi, M.; Branduardi, D.; Camilloni, C.; Bussi, G. PLUMED 2: New feathers for an old bird. *Computer Physics Communications* **2014**, *185*, 604–613.

(33) Bennett, C. H. Efficient estimation of free energy differences from Monte Carlo data. *Journal of Computational Physics* **1976**, *22*, 245–268.
