# Supplementary material for: Ice Recrystallization Inhibition by Amino Acids: The Curious Case of Alpha- and Beta-Alanine
Source: J Phys Chem Lett. 2022 Mar 3;13(9):2237–44. doi: 10.1021/acs.jpclett.1c04080 (PMC9007522; doi:10.1021/acs.jpclett.1c04080)
Supplement: Supplementary file 2 — jz1c04080_si_002.pdf [file jz1c04080_si_002.pdf]

jz-2021-04080u.R1

Name: Peer Review Information for "Ice Recrystallisation Inhibition by Amino Acids: The Curious Case of Alpha- and Beta-Alanine"

First Round of Reviewer Comments

Reviewer: 1

Comments to the Author

1. The work by Warren et al. reports on the IRI activity of the amino acid alanine and its isomer betaine. Apart from IRI experiments, MD simulations have been performed to identify a possible molecular mechanism. The manuscript is well written and it is interesting that the amino acid alanine shows IRI activity although under very unique experimental conditions. The simulation studies are also interesting and could present at advance if verified with other molecules with known IRI properties. However, overall, the manuscript lacks novelty and seems to better suited for a more specialized journal (e.g. Langmuir). Beyond that there is a major concern that should be addressed.

2. The immediate significance of the advances presented in this manuscript are not clear. IRI activity of alanine is interesting but given that it shows no activity in PBS buffer means it will not advance cryopreservation. The simulations will be beneficial for a specialized audience.

3. Major points and Technical suggestions

The main concern is that the IRI data was observed under conditions that are atypical for the field and which prevents comparison with other literature data. The accompanying simulations are interesting but it seems that the two observables are not necessarily connected and require further experimental evidence. Scenarios like differences in the hydration shell of the two molecules (e.g. shown by extensive work by the Havenith lab) or aggregation that would fit into Robert Ben's explanation model were not considered and should be addressed.

The authors could easily check experimentally whether their proposed mechanism is in fact due to differences in the engulfment/overgrowth in the ice. A simple essay of how much sample gets incorporated could be performed with a cold-finger and by then checking the concentration of the compounds in the molten ice sample.

p.4 l.23 The statement assessing amino acids is not correct as only one AA was measured.

p.4 l.34 The authors report that in PBS buffer, the standard for IRI measurements, no IRI activity is observed. This is concerning and has to be explained further and not just by citing a work that partially addresses the topic. Moreover, since the Sucrose assay again showed no activity in the case of alanine. If

no activity is observed in PBS buffer the compound will also not be beneficial for cryopreservation application since cells are typically stored in PBS or DPBS buffer.

The fact that 10 mM NaCl causes activity should be explained (possible structural changes, aggregate formation), maybe simulations can help. It is further not helpful when standard conditions are neglected that allow comparison of IRI data across labs to report conditions where activity is suddenly observed without an explanation. Could betaine suddenly show high activity under different conditions?

p.5 l. 15 Statements should we weakened as alanine remains a weak/no inhibitor under experimental conditions that are not comparable to the mentioned reported inhibitors. Furthermore, smaller inhibitors such as surfactants showed IRI activity at even lower (micromolar) concentrations (e.g. Fan et al. 2020 Langmuir).

p.7 l. 45 Consisting with experiments…. This statements seem incorrect since no ice shaping was observed. If simulations see an effect then this should also be measurable in the experiments which does not seem to be the case here. Please explain!

Reviewer: 2

Comments to the Author

In this article, authors have presented “Ice Recrystallisation Inhibition by Amino Acids: The Curious

Case of Alpha- and Beta-Alanine”. They have used both experimental and computational studies on the system. Experimental results are certainly interesting. However, the computational part of the paper where authors have tried to explain their experimental results are not convincing. I will list my concerns over the computational part:

(1) Authors have mentioned that “The growth rate is directly proportional to the IRI activity”. Authors should provide a comprehensive explanation of this statement. In my opinion, IRI activity is a long time phenomena. Therefore, a slightly slower growth rate does not really tell anything about IRI activity. Please explain this statement with proper references.

(2) As authors have mentioned that many of the simulation trajectories have shown overgrown scenario for both the systems within this limited system size and timescale, therefore, in real systems all of the system will be eventually overgrown given longer timescale. Therefore, mere slowing down does not explain the IRI. I think it has to stop the growth by some means.

(3) The closer match of the hydrogen bonding atoms in beta alanine and ice is linked to the overgrowth. If anything, in the literature, the opposite trend is reported for proteins. I think just because it works here, one can not say that this is the reason. One needs to come up with some explanations. May be a calculation of solvation energy can provide better understanding. Similar kind of calculations are reported for the brine rejection phenomena with different ions. Author may look into the same.

Therefore, while the experimental result is interesting, the computational part does not provide a clear explanation of the results. I believe that authors might put some more efforts in the computational part to make it a significant contribution to the field.

Author's Response to Peer Review Comments:

21 January 2022

Dear Prof. Editor,

we thank you for handling our manuscript. We are genuinely thankful for having received some excellent comments from the Reviewers. We have thoroughly addressed all the issues raised by the Reviewers and put together a substantially improved revised version of the manuscript. Most prominently:

- We have performed additional experiments, which show that alpha-alanine is active in PBS buffer as well – within a range of effective salts concentrations similar to what we have used for our original splat assays.
- We have performed additional simulations which allowed us to compute both the hydration index and the solvation free energy of alpha- and beta-alanine. The findings are consistent with our original claims and strengthen even further our molecular description of the ice-alanine interactions

Our replies to the Reviewers' comments, together with the description of the corresponding changes made in the article (highlighted in purple) are reported in the following pages. We hope that this revised version of the manuscript would now be acceptable for publication in the Journal of Physical Chemistry Letters.

Best regards,

Dr. Gabriele C. Sosso - on behalf of all the authors.

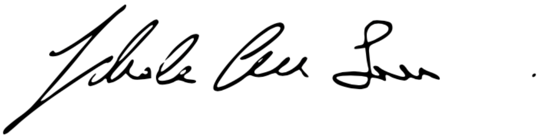

Note: references in **green** can be found at the end of this document.

- Response to Reviewer #1 –

Recommendation: While the work is good and publishable, a more appropriate journal is recommended such as Langmuir, JPCB

Our reply: We thank the Reviewer for their positive assessment of the quality of our work. In the process of addressing the Reviewer's comments (see below) we have performed additional experiments as well as additional computational analysis. We believe that these new results highlight the novelty and significance of our work, with an emphasis on the suitability of this manuscript to be published in the Journal of Physical Chemistry Letter in terms of both originality and timeliness.

Reviewer's comment: *1) The work by Warren et al. reports on the IRI activity of the amino acid alanine and its isomer betaine. Apart from IRI experiments, MD simulations have been performed to identify a possible molecular mechanism. The manuscript is well written, and it is interesting that the amino acid alanine shows IRI activity although under very unique experimental conditions. The simulation studies are also interesting and could present at advance if verified with other molecules with known IRI properties. However, overall, the manuscript lacks novelty and seems to better suited for a more specialized journal (e.g. Langmuir). Beyond that there is a major concern that should be addressed.*

Our reply: Although the IRI of betaine was investigated in our experimental controls (Figure S1), we would like to clarify that the amino acids primarily investigated in this study were alpha-alanine and its isomer beta-alanine: all the analysis in the main text pertains to these two molecules, and not to betaine.

In terms of the manuscript's novelty, we wish to highlight that this work is the first to report that individual amino acids can display potent IRI activity. This is itself a novel finding, amplified by the fact that these molecules are also incredibly small; indeed, to the best of our knowledge, they are smaller than any other inhibitors reported. This includes various small molecules reported in the work of Robert Ben and colleagues, which can have as few as around 40 atoms (250-300 Da) **[1,2]**.

In terms of the validity of the simulations, we would like to point out that our methodology has been applied with excellent results (particularly in terms of the correlation with the experimentally observed IRI activity) to polymers **[3]** as well as small peptides **[4]**. We have added a statement to this effect in the revised version of the manuscript [page 7]: "This computational methodology has been validated extensively and results obtained via this set up have shown excellent correlation with the experimentally observed IRI activity of both polymers[34] and small peptides[35]".

As such, we are very confident in the robustness of our simulations, which show an entirely different IRI mechanism if compared to most biological or polymeric systems. The computational aspect complements nicely the experimental work and yields a comprehensive picture of the IRI activity of alpha-alanine and beta-alanine. Given the pace at which the field is evolving, these findings represent an urgent piece of knowledge that, we feel, fits very well with the scope of the Journal of Physical Chemistry Letter.

We address the Reviewer's point re: the uniqueness of the experimental conditions below.

Reviewer's comment: *2) The immediate significance of the advances presented in this manuscript are not clear. IRI activity of alanine is interesting but given that it shows no activity in PBS buffer means it will not advance cryopreservation. The simulations will be beneficial for a specialized audience.*

Our reply: We acknowledge that, in principle, the low activity of alpha-alanine in PBS buffer represents a limitation of its cryopreservation applications - at least in the context of mammalian cells, see below. This is why we have avoided any claims that alpha-alanine itself would be an excellent cryoprotectant. Instead, the manuscript highlights the potential of amino acids – very small molecules – to inhibit ice growth and puts forward a novel mechanism of inhibition for these materials. Hence, the work identifies a new class of IRI-active materials that may be investigated for cryoprotective applications, emphasised via the addition of the following sentence to the revised version of the manuscript [page 15]: "We have also brought to attention amino acids as a new class of IRI-active material [...]"

We have also highlighted a future avenue for this work, indeed one which we are already pursuing, which is to identify amino acids that can inhibit IR effectively at high salt concentrations. We have added the following line in the revised version of the manuscript to this effect [page 16]: "In light of the limited IRI activity of α-alanine under high salt concentrations, the identification of saline-stable inhibitors represents a focal point for future work."

In terms of the Reviewer's concern regarding atypical experiments conditions (in both comments 2 and 3) we note that similar conditions have been used in other cases, such as in the work by Balcerzak et. al., **[1]** who report the activity of a new class of inhibitors - lysine-based surfactants and quaternary ammonium salts – via the same "splat" assay using 0.5 mg/ml (9 mM) saline instead of PBS, which they rationalise as follows: *"A 0.5 mg/mL NaCl solution was used rather than a PBS solution (which is typically used in this assay) as the compounds were more soluble in the NaCl solution. The use of 0.5 mg/mL NaCl has been previously utilized in this assay and is very effective at excluding false-positive recrystallization effects. This solution is similar to PBS and physiological conditions."* To highlight the use of these conditions in other bodies of work, we have added the following statement to the manuscript [page 4]: "We also note that, while this assay is usually performed in PBS, the use of saline solution for IRI measurements is not uncommon in the field.[24,25]"

However, in order to address the Reviewer's comment properly, we have performed additional experiments, which show that alpha-alanine does remain active in 10 mM phosphate buffer, whilst it is only weakly active in 100 mM NaCl solution. This evidence is suggestive that the IRI activity of alpha-alanine is sensitive to high concentrations of salts such as those present in PBS (136 mM NaCl), rather than specific components of the buffer. In addition to updating Figure S1 in the manuscript (see below), we have modified the main text in reference to this new data as follows [page 4]: "We also found that α-alanine remains active in 10 mM phosphate buffer, but not under higher concentrations of saline (100 mM NaCl) (Figure S1), hence we suggest that the IRI activity of this system is sensitive to high concentrations of salt, rather than specific components of PBS solution."

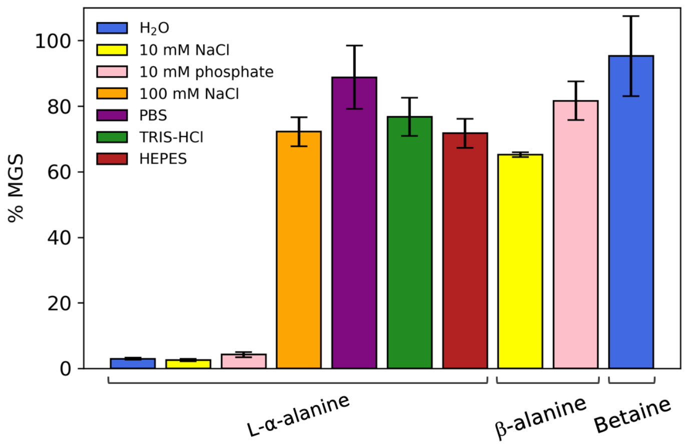

Figure S1: Ice recrystallisation inhibition of 100 mM L-α-alanine, β-alanine or betaine under different solution conditions. Each data point is reported relative to the MGS of the solution (without amino acid) alone, except in the case of pure water (as pure water does not ripen). A negative control of 100 mM L-α-alanine in water (as this amino acid does not slow ice growth) was used in this case.

In line with this finding, we note that there are a large number of biological materials that are handled and stored in low-salt media, with total salt concentrations below 100 mM. These media include 2xYT media **[5]**, TB media **[6]**, and M9 media **[7]** used in the culture of bacteria strains, and MS media **[8]** used in plant cell culture. Given the need to cryopreserve bacterial and plant cell stocks for both research and industrial purposes, a range of applications could stand to benefit from this work. We have modified the manuscript accordingly, to emphasise these applications over mammalian cell cryopreservation [page 5]: "In the knowledge that α-alanine is inactive at high salt concentrations (100 mM and above), we note that the cryoprotective applications

of α-alanine specifically may be limited to cases where biological materials are stored in low-salt buffers. However, we highlight that there are a number bacterial and plant growth media which satisfy these minimal salt requirements (e.g., 2xYT,[29]M9,[30] TB[31] and MK[32] media), and such materials require effective cryopreservation for both industrial and research applications"

We also wanted to highlight the significance of the simulations: whilst the technical details of the methodology might appeal to computational scientists only, the findings, particularly the mechanism by which these amino acids manage to slow the growth of ice, are of great importance to the field as a whole. The debate on the molecular details at the heart of the IRI activity of different classes of compounds, from proteins to polymers to inorganic materials, has been raging for the best part of a decade, and this work represents a key, timely contribution to this debate.

Reviewer's comment: *3) The main concern is that the IRI data was observed under conditions that are atypical for the field and which prevents comparison with other literature data. The accompanying simulations are interesting, but it seems that the two observables are not necessarily connected and require further experimental evidence. Scenarios like differences in the hydration shell of the two molecules (e.g., shown by extensive work by the Havenith lab) or aggregation that would fit into Robert Ben's explanation model were not considered and should be addressed. The authors could easily check experimentally whether their proposed mechanism is in fact due to differences in the engulfment/overgrowth in the ice. A simple essay of how much sample gets incorporated could be performed with a cold-finger and by then checking the concentration of the compounds in the molten ice sample.*

Our reply: We have discussed in the point above the robustness of our findings regarding different experimental conditions.

In terms of the connection between experiments and simulations, the former measure the mean grain size (MGS) of the ice crystals, whilst the simulations quantify the efficiency of a given compound to slow down the kinetic of ice growth (at the experimental temperature). Whilst the recrystallization of ice is, strictly speaking, a form of Ostwald ripening that clearly lies far beyond the reach of molecular simulations, the kinetics of ice growth are directly related to the MGS of crystals. Proof of the connection between MGS and ice growth kinetics is given by our recent body of work on the IRI activity of polymers as well as small peptides **[3,4]**, where we have demonstrated that – in the same fashion as the results reported in this work – the extent to which a given compound manages to slow down the growth of the ice crystals correlates spectacularly well with the MGS observed experimentally. This is even more striking in the context of this work, as the difference between the IRI activity of alpha- vs beta-alanine is stark but still much more subtle than that of, say, PVA vs PEG. And yet, our simulations – chiefly thanks to the number of statistically independent simulations we have performed, managed to capture the experimental trend.

However, following the Reviewer's suggestion regarding the work of Robert Ben, we have now computed a hydration index **[9]** of both alpha- and beta-alanine via MD simulations. The details of this method are included in the revised Supplementary Information. The hydration index of alpha- and beta-alanine were calculated as 0.152 ± 0.023 and 0.162 ± 0.021 molecules/Å^3 (mean ± standard deviation over 10 ns simulations), respectively. This small difference suggests that the de-solvation of beta-alanine might results in a slightly greater entropic gain if compared to alpha-alanine. Thus, it appears that not only beta-alanine interact more strongly with the ice surface in terms of hydrogen bonding, but it also features a slightly more favourable entropic gain upon binding. In order words, this analysis confirms that the interaction between beta-alanine and ice is stronger than that between ice and alpha-alanine. The IRI mechanism thus has nothing to do with interaction strength (albeit of course alpha-alanine still needs to be able to interact with the ice surface) but with the structural features of these amino acids with respect to the ice lattice – as discussed in detail in the manuscript. In fact, we have also computed the solvation free energy of both species – see our answer to Reviewer 2, comment #3, which consolidate even further this computational result.

The discussion re: the hydration index as been incorporated into the revised version of the manuscript [page 12] as follows: "Given the difference in the hydrogen bonding capacities of α- and β-alanine, we investigated differences in the solvation shells of these two molecules. We determined the hydration index, based on the

definition provided by Tam et al.,[19] to be 0.152 ± 0.023 and 0.162 ± 0.021 molecules/Å^3, for α- and β-alanine respectively. This small difference suggests a marginally greater entropic gain associated with the desolvation of β-alanine compared to α-alanine."

We also acknowledge the effectiveness of ice affinity purification using a cold-finger to study this property in the case of antifreeze proteins and ice-binding polymers. However, as the amino acids presented here do not show high affinity for ice surfaces (shown by ice shaping measurements, Figure S2) we do not believe this technique would be appropriate in this case. We are actively exploring other means of tackling these and similar situations in the future.

p.4 l.23 The statement assessing amino acids is not correct as only one AA was measured. We acknowledge that this statement is unclear given that alpha- and beta-alanine, although different amino acids, are both isomers of alanine. For clarification, we have modified the following line in the manuscript [page 4]: "We began by assessing the ability of α- and β-alanine to inhibit IR using the "splat" cooling assay".

p.4 l.34 The authors report that in PBS buffer, the standard for IRI measurements, no IRI activity is observed. This is concerning and has to be explained further and not just by citing a work that partially addresses the topic. Moreover, since the Sucrose assay again showed no activity in the case of alanine. If no activity is observed in PBS buffer the compound will also not be beneficial for cryopreservation application since cells are typically stored in PBS or DPBS buffer. The fact that 10 mM NaCl causes activity should be explained (possible structural changes, aggregate formation), maybe simulations can help. It is further not helpful when standard conditions are neglected that allow comparison of IRI data across labs to report conditions where activity is suddenly observed without an explanation. Could betaine suddenly show high activity under different conditions?

<u>Our reply:</u> Although this comment is valid for most mammalian cells, there are a range of biological materials which are handled in low-salt media and require cryopreservation, as discussed in our response to Reviewer's comment #1.

In addition, further experimental work has shown that alpha-alanine is also highly active in 10 mM phosphate buffer and that IRI activity is lost at higher concentrations of NaCl (100 mM, Figure S1). We therefore show that it is not 10 mM NaCl specifically that *causes* activity, but that the IRI activity of alpha-alanine is dependent and sensitive to the total amount of salt present. See our response to Reviewer's comment #1 for additional details. For completeness, we also measured the IRI of activity of beta-alanine in 10 mM phosphate, observing no IRI activity. All these results have been added to Figure S1 in the manuscript, in addition to the comments mentioned in our reply to Reviewer's comment #2.

p.5 l. 15 Statements should we weakened as alanine remains a weak/no inhibitor under experimental conditions that are not comparable to the mentioned reported inhibitors. Furthermore, smaller inhibitors such as surfactants showed IRI activity at even lower (micromolar) concentrations (e.g. Fan et al. 2020 Langmuir).

<u>Our reply:</u> We acknowledge that the difference in experimental conditions makes it difficult to directly compare the IRI activity to other materials evaluated by other research groups. In the manuscript, we have made attempts to ensure that where a comparison is made, the experimental conditions are as similar as possible, other than the solution composition itself. We mention that the activity of these materials is moderate in comparison to others such as PVA, giving precise numbers to illustrate this point more precisely. We have also removed the statement on p.5 l.15 in line with the reviewer's comments, and replaced it instead with the following, citing work where the "splat" cooling assay was performed in ~10 mM NaCl rather than PBS **[1]** [page 5]: "This level of activity can be considered moderate in comparison to the most potent IRI-active materials such as poly-vinyl alcohol (PVA),[26] but on the same magnitude as other small molecules tested under similar conditions.[24]"

We would like also to highlight that, in the work referenced by the Reviewer **[10]**, the "splat" assay was performed under atypical conditions (annealing for 60 minutes at -15ºC) and IRI activity values were reported

using an entirely different metric: absolute mean grain size (MGS), rather than % MGS relative to a control. As pointed out by the Reviewer, such factors make it difficult to compare to the data presented in this manuscript. One metric that can be easily compared are the molecules' size, and we note that the surfactants are not smaller than the amino acids reported here, as suggested by the reviewer: the smallest surfactant in the referenced work contains over 40 atoms (~290 Da) compared to a composition of 13 atoms (~90 Da) for alpha-alanine.

p.7 l. 45 Consisting with experiments…. These statements seem incorrect since no ice shaping was observed. If simulations see an effect then this should also be measurable in the experiments which does not seem to be the case here. Please explain!

Our reply: This statement was certainly unclear and has been amended [page 7]: "In the simulations concerning the primary and secondary prismatic planes, we found that the presence α-alanine resulted in slower rates of growth compared to β-alanine (Figure 2b), consistent with our experimental % MGS data. The basal fronts displayed similar rates of growth in the presence of either α- or β-alanine, although we note that in the context of IRI, growth (inhibition) at the prismatic fronts is considered of greater relevance due to the rapid growth rates observed from these faces relative to the basal face.[17,40]"

To clarify the point here in greater detail, we had intended to communicate that the slower growth rates were observed for alpha-alanine compared to beta-alanine, for the primary and secondary prismatic planes only. Comparing the growth rates for the basal plane simulations, we do not see a significant difference between simulations of alpha- and beta-alanine. For IRI activity, inhibition of basal plane growth is not considered nearly as relevant or indeed a requirement, as the basal plane displays slower kinetics than prismatic planes. This is also captured in our simulations. Overall, this data is consistent with results from the "splat" cooling assay (greater ice growth observed for beta-alanine than for alpha-alanine). Viewing the data for alpha- and beta-alanine independently, the growth rates are broadly similar across the primary and secondary prismatic planes. This result is consistent with ice shaping experiments, which can be used to identify binding and inhibition at specific ice faces, most often the prismatic and pyramidal planes **[11]**.

- Response to Reviewer #2 -

Recommendation: This paper may be publishable, but major revision is needed; I would like to be invited to review any future revision. […] In this article, authors have presented "Ice Recrystallisation Inhibition by Amino Acids: The Curious Case of Alpha- and Beta-Alanine". They have used both experimental and computational studies on the system. Experimental results are certainly interesting. However, the computational part of the paper where authors have tried to explain their experimental results are not convincing. […] Therefore, while the experimental result is interesting, the computational part does not provide a clear explanation of the results. I believe that authors might put some more efforts in the computational part to make it a significant contribution to the field.

Our reply: We thank the Reviewer for their comments and their assessment of our work. Following the Reviewer's suggestions, we have performed additional simulations aimed at quantifying both the extent of the solvation shell for alpha- and beta-alanine, as well as their solvation free energy. We are happy to report that the results are consistent with our previous findings and indeed serve to strengthen our original claims.

Reviewer's comment: *1) Authors have mentioned that "The growth rate is directly proportional to the IRI activity". Authors should provide a comprehensive explanation of this statement. In my opinion, IRI activity is a long time phenomena. Therefore, a slightly slower growth rate does not really tell anything about IRI activity. Please explain this statement with proper references.*

Our reply: The Reviewer is certainly correct in pointing out the gap between what the experiments measure and what the simulations quantify. Ice recrystallisation inhibition occurs primarily via Ostwald ripening. In the Ostwald ripening model, water molecules migrate from the surface of small ice crystals to larger crystals,

diffusing through the bulk water and a pre-ordered interfacial layer (or quasi-liquid layer) surrounding each crystal. As larger crystals have a lower surface area to volume ratio, they have a lower interfacial energy. Overall, the process therefore results in an increase in the average grain size and a net reduction in free energy **[12,13]**.

The experimental reality of the ice recrystallisation is that there is always a fraction of un-frozen, supercooled water at the boundaries between ice crystals. In order for the larger ice crystals to grow, water molecules must transfer from this bulk fraction (liquid) onto the surface of a growing crystal. In our simulations, we are measuring the kinetics of this process – the growth rate of ice – in presence of amino acids molecules. Although the simulations do not capture every step of ice recrystallisation (e.g. transfer from ice to the bulk, or diffusion through the bulk fraction), the transfer from supercooled liquid to ice typically represents the slowest, limiting step of the overall process in presence of ice recrystallisation inhibitors and given a high ice volume fraction present in "splat" cooling assay **[14]**. Our measurements are therefore proportional to the IRI activity determined using this experimental assay.

Indeed, the results obtained using this computational methodology have been found to directly correlate with the experimental IRI activity data for a number of systems, including the polymer poly-vinyl alcohol (PVA), of varying degrees of polymerisation [3] and a range of small peptide molecules [4]. An additional argument to support this point can be found in our reply to Reviewer 1, Comment #3, and we provide here the relevant changes to the manuscript [page 7]: "We note that while these simulations do not fully capture the complex recrystallisation process (Ostwald ripening) in its entirety, the transfer of water molecules from the supercooled liquid fraction at the grain boundaries to the surface of a growing crystal is a fundamental step in ice recrystallisation. Our simulations capture the kinetics of this process (i.e. the growth rate), which is thus directly proportional to the rate of ice recrystallisation represented by the % MGS metric. This computational methodology has been validated extensively and results obtained via this set up have shown excellent correlation with the experimentally observed IRI activity of both polymers[38] and small peptides.[39]"

Reviewer's comment: *2) As authors have mentioned that many of the simulation trajectories have shown overgrown scenario for both the systems within this limited system size and timescale, therefore, in real systems all of the system will be eventually overgrown given longer timescale. Therefore, mere slowing down does not explain the IRI. I think it has to stop the growth by some means.*

Our reply: It is true that both systems show cases where the (alpha/beta-)alanine molecule becomes overgrown. In total, we see 13/60 trajectories overgrown for alpha-alanine, compared to 32/60 for beta-alanine for simulations with one (alpha/beta-)alanine molecule; for simulations with two amino acid molecules, these numbers are 18/60 and 42/60, for alpha- and beta-alanine respectively. The slower kinetics we observe for beta-alanine must also considered in light of these outcomes. As beta-alanine is frequently overgrown, the concentration of beta-alanine in ice will increase concomitantly with ice growth. Alpha-alanine is only occasionally overgrown, and more often forced along the growth (in this case -z) axis the end of the simulation cell, slowing the rate of growth in the process. The effective concentration of alpha-alanine therefore increases in bulk system at the grain boundaries, relative to beta-alanine in an equivalent system. As more alpha-alanine accumulates in this eutectic, the effect compounds – growth is slowed further as the concentration of alpha-alanine, and therefore its surface coverage, increases. At longer timescales, given a highly saturated bulk phase, all growth could conceivably be arrested. We have added a brief statement to explain this in the manuscript [page 10]: "We also suggest that overgrowth of (α/β-)alanine molecules has a compounding effect, as it sequesters the amino acids in the ice fraction. Consequently, the effective concentration of α-alanine at the grain boundaries could increase over time relative to β-alanine, further slowing ice growth via greater surface coverage."

We would also like to point out that these simulations represent a substantial step forward in relation to the previous computational literature, in which - in most cases – the IRI activity of a given compound has been estimated on the bases of its interaction with fixed, pristine, static ice surfaces, in some cases even in the absence of the water phase. Our simulations, which feature growing, rough, moving ice surfaces at the same

supercooling investigated experimentally, manage to capture kinetics effect which correlate very well indeed with the experimental outcomes of "splat" cooling assays.

Reviewer's comment: *3) The closer match of the hydrogen bonding atoms in beta alanine and ice is linked to the overgrowth. If anything, in the literature, the opposite trend is reported for proteins. I think just because it works here, one can not say that this is the reason. One needs to come up with some explanations. May be a calculation of solvation energy can provide better understanding. Similar kind of calculations are reported for the brine rejection phenomena with different ions. Author may look into the same.*

Our reply: The Reviewer is exactly right in stressing the difference between the IRI mechanism we have observed for these small amino acids compared to that reported in the case of e.g. antifreeze proteins. In fact, we believe that this stark difference is a major aspect contributing to the novelty and timeliness of our results. Following the suggestion of the Reviewer, we have computed the solvation free energy of both alpha- and beta-alanine, via the Bennett acceptance ratio (BAR) method **[15]**. The solvation free energy ΔG_{Solv} has been computed as: $\Delta G_{Solv} = \Delta G_{Coulomb} + \Delta G_{vdW}$, where $\Delta G_{Coulomb}$ and ΔG_{VdW} correspond to the contributions of long-range electrostatic (Coulomb) interactions and van der Waals (vdW) interactions, respectively. Details of these calculations are included in the revised version of the Supplementary Information. Crucially, we have verified the robustness of our results to the choice of the paths utilised to switch off long-range electrostatic interactions as well as van der Waals interactions, ensuring the reversibility of the process and thus the absence of hysteretic effects.

The solvation free energy of alpha-alanine is -36.9 ± 0.1 kcal/mol, whilst the solvation free energy of beta-alanine is -43.5 ± 0.2 kcal/mol. Our results are in line with those previously reported for (the zwitterionic form of) alpha-alanine in Ref. **[16]** by using the OPLS-AA / TIP4P/Ice force fields. The fact that beta-alanine is characterised by a larger ΔG_{Solv} if compared with alpha-alanine is consistent with our observation that beta-alanine has a greater potential to form hydrogen bonds with the water/ice phase, as well as with the analysis of the hydration shell (which contains on average more water molecules than alpha alanine, as illustrated by the hydration index discussed in our reply to Reviewer 1, Comment #3). These findings strengthen the hypothesis that beta-alanine interacts with ice more strongly than alpha: it is instead the structural fit of these two molecules re: the ice lattice which determines to which extent they get – on average – overgrown. We have made the following additions to the manuscript in light of these findings [page 12]: "To consolidate these results, we also computed the solvation free energy, ΔG_{solv}, for both species via MD simulations using the Bennett Acceptance Ratio method.[45] The solvation energies of α- and β-alanine were found to be −36.9 ± 0.1 and −43.5 ± 0.2 kcal/mol, respectively, in line with solvation energies previously reported.[46] The greater solvation energy of β-alanine compared to α-alanine is consistent with both hydrogen bonding and hydration index data. These results at first appear to counter intuition: β-alanine can form a greater number of hydrogen bonds with ice thanα-alanine and stands to benefit from a larger entropic gain upon binding, yet it is less effective at inhibiting ice growth. We instead suggest that a stronger interaction with the ice front, confirmed by the aforementioned computational analyses, could be detrimental to the IRI activity of these small molecules as it increases the likelihood of becoming overgrown"

The reviewer points out that the opposite trends is found for proteins, and we certainly agree that alpha- and beta-alanine are characterised by a rather unique IRI mechanism. However, we believe that the balance between (a.) the interactions between the ice phase and a given IRI agent and (b.) the effect of the morphology of the latter, whether this implies a large effective volume of the IRI agent at the ice/water interface (see our work on PVA [3]) or structural fit to the ice lattice (as in this case), is a subtle one. As brilliantly summarised in Ref. **[17]** in the case of anti-freeze proteins: *"At temperatures below the freezing point these enthalpic changes will only be partially compensated by the formation of protein-ice hydrogen bonds and a possible entropic gain due to the release of solvation water. This argument, however, does not trivialize the importance of the existent correct spacing of polar residues. Polar group matching significantly reduces the loss of ice hydrogen bonds and their concurrent unfavorable enthalpic consequence and will lead to specific binding,*

even if overall the enthalpic contribution of the protein-ice hydrogen bonding to the Gibbs energy of adsorption were positive".

A statement has been added to the main text to reflect this notion [page 16]: "We note that the trends observed here with respect to ice binding may be contrary to those reported in the literature for e.g. antifreeze proteins,[15] highlighting the different structural determinants at play in small molecule IRIs."

References

[1] A. K. Balcerzak, M. Febbraro, and R. N. Ben, *The Importance of Hydrophobic Moieties in Ice Recrystallization Inhibitors*, RSC Adv. **3**, 3232 (2013).

[2] C. J. Capicciotti, R. S. Mancini, T. R. Turner, T. Koyama, M. G. Alteen, M. Doshi, T. Inada, J. P. Acker, and R. N. Ben, *O-Aryl-Glycoside Ice Recrystallization Inhibitors as Novel Cryoprotectants: A Structure–Function Study*, ACS Omega **1**, 656 (2016).

[3] F. Bachtiger, T. R. Congdon, C. Stubbs, M. I. Gibson, and G. C. Sosso, *The Atomistic Details of the Ice Recrystallisation Inhibition Activity of PVA*, Nat. Commun. **12**, 1 (2021).

[4] C. A. Stevens, F. Bachtiger, X.-D. Kong, L. A. Abriata, G. C. Sosso, M. I. Gibson, and H.-A. Klok, *A Minimalistic Cyclic Ice-Binding Peptide from Phage Display*, Nat. Commun. **12**, 2675 (2021).

[5] A. C. Miklos, M. Sarkar, Y. Wang, and G. J. Pielak, *Protein Crowding Tunes Protein Stability*, J. Am. Chem. Soc. **133**, 7116 (2011).

[6] A. Arabi Shamsabadi, M. Sharifian Gh., B. Anasori, and M. Soroush, *Antimicrobial Mode-of-Action of Colloidal Ti3C2Tx MXene Nanosheets*, ACS Sustain. Chem. Eng. **6**, 16586 (2018).

[7] Q. Wang, A. Alowaifeer, P. Kerner, N. Balasubramanian, A. Patterson, W. Christian, A. Tarver, J. E. Dore, R. Hatzenpichler, B. Bothner, and T. R. McDermott, *Aerobic Bacterial Methane Synthesis*, Proc. Natl. Acad. Sci. **118**, (2021).

[8] J. C. Bettoni, R. Bonnart, and G. M. Volk, *Challenges in Implementing Plant Shoot Tip Cryopreservation Technologies*, Plant Cell Tissue Organ Cult. PCTOC **144**, 21 (2021).

[9] R. Y. Tam, S. S. Ferreira, P. Czechura, J. L. Chaytor, and R. N. Ben, *Hydration Index—A Better Parameter for Explaining Small Molecule Hydration in Inhibition of Ice Recrystallization*, J. Am. Chem. Soc. **130**, 17494 (2008).

[10] Q. Fan, Y. Gao, C. Zhu, J. Liu, L. Zhao, J. Mao, S. Wu, H. Xue, J. S. Francisco, X. C. Zeng, and J. Wang, *Unraveling Molecular Mechanism on Dilute Surfactant Solution Controlled Ice Recrystallization*, Langmuir **36**, 1691 (2020).

[11] T. Berger, K. Meister, A. L. DeVries, R. Eves, P. L. Davies, and R. Drori, *Synergy between Antifreeze Proteins Is Driven by Complementary Ice-Binding*, J. Am. Chem. Soc. **141**, 19144 (2019).

[12] C. Budke, C. Heggemann, M. Koch, N. Sewald, and T. Koop, *Ice Recrystallization Kinetics in the Presence of Synthetic Antifreeze Glycoprotein Analogues Using the Framework of LSW Theory*, J. Phys. Chem. B **113**, 2865 (2009).

[13] R. L. Sutton, A. Lips, G. Piccirillo, and A. Sztehlo, *Kinetics of Ice Recrystallization in Aqueous Fructose Solutions*, J. Food Sci. **61**, 741 (1996).

[14] P. R. Rios, F. Siciliano Jr, H. R. Z. Sandim, R. L. Plaut, and A. F. Padilha, *Nucleation and Growth during Recrystallization*, Mater. Res. **8**, 225 (2005).

[15] C. H. Bennett, *Efficient Estimation of Free Energy Differences from Monte Carlo Data*, J. Comput. Phys. **22**, 245 (1976).

[16] J. Chang, A. M. Lenhoff, and S. I. Sandler, *Solvation Free Energy of Amino Acids and Side-Chain Analogues*, J. Phys. Chem. B **111**, 2098 (2007).

[17] F. D. Sönnichsen, C. I. DeLuca, P. L. Davies, and B. D. Sykes, *Refined Solution Structure of Type III Antifreeze Protein: Hydrophobic Groups May Be Involved in the Energetics of the Protein–Ice Interaction*, Structure **4**, 1325 (1996).
